# ADAMTS4 mediates LPS‐induced cardiac injury and myocardial fibrosis through proteolytic cleavage of TSP1

**DOI:** 10.1002/ctm2.70769

**Published:** 2026-08-05

**Authors:** Zhe‐Wei Zhang, Shi‐Yan Wan, Wen Li, Xiao Lu, Rong Chen, Xin Xing, Wei Lai, Shu‐Yang Sheng, M. M. Mosebetsi, Qing‐Tao Meng, Zi‐Long Lu, Di Fan, Wan‐Li Jiang

**Affiliations:** ^1^ Department of Thoracic Surgery Renmin Hospital of Wuhan University Wuhan China; ^2^ Department of Emergency Renmin Hospital of Wuhan University Wuhan China; ^3^ Department of Anesthesiology Renmin Hospital of Wuhan University Wuhan China; ^4^ Department of Cardiology Zhongnan Hospital of Wuhan University Wuhan China

**Keywords:** ADAMTS4, cardiac fibrosis, cardiac function, inflammation, TSP1

## Abstract

Septic cardiomyopathy is characterized by acute cardiac dysfunction, while sustained inflammatory and extracellular matrix remodeling may contribute to subsequent myocardial fibrosis and impaired cardiac recovery. A disintegrin and metalloproteinase with thrombospondin motifs 4 (ADAMTS4) has been linked to fibrotic remodelling, but its role in septic cardiac injury remains unclear. Here, we show that *Adamts4* is upregulated in LPS‐induced septic mouse hearts and is predominantly induced in cardiac fibroblasts. Using three fibroblast‐lineage Adamts4 knockout models, we demonstrate that Adamts4 deletion attenuates LPS‐induced cardiac dysfunction, inflammation and fibrosis, whereas Tcf21‐lineage fibroblast *Adamts4* overexpression aggravates these changes. Mechanistically, ADAMTS4 interacts with thrombospondin‐1 (TSP1) and promotes its catalytic activity‐dependent proteolytic processing. The 236–246 amino acid region of TSP1 is required for fragment generation and fibroblast activation. TSP1 processing further activates TGF‐β/Smad and NF‐κB signalling, promoting myofibroblast activation and extracellular matrix deposition. These findings identify ADAMTS4 as a fibroblast‐centred driver of septic cardiac fibrosis and suggest that targeting the ADAMTS4‐TSP1 axis may offer therapeutic potential for septic cardiomyopathy.

**Key Points:**

- ADAMTS4 is induced predominantly in cardiac fibroblasts after LPS challenge
- Fibroblast‐lineage Adamts4 deletion attenuates LPS‐induced cardiac dysfunction and fibrosis
- The TSP1 236‐246 aa region is required for efficient ADAMTS4‐dependent processing
- ADAMTS4‐dependent TSP1 processing activates TGF‐β/Smad and NF‐κB signalling in fibroblasts.

## Graphical abstract

• ADAMTS4 is induced primarily in cardiac fibroblasts during sepsis.

• ADAMTS4 cleaves TSP1 at the 236–246 aa functional region.

• TSP1 cleavage activates TGF‐β/Smad and NF‐κB signalling in fibroblasts.

• Targeting ADAMTS4 reduces fibroblast activation and cardiac fibrosis.

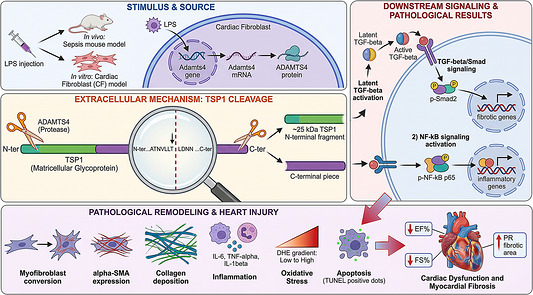

## 1 INTRODUCTION

Sepsis is defined as infection‐related organ dysfunction caused by an imbalanced host response^1^ and continues to impose a substantial clinical burden, especially in intensive care units, because of its high incidence and mortality.^2^ Among its complications, septic cardiomyopathy (SCM) represents a reversible but clinically important myocardial dysfunction that occurs in a large proportion of septic patients and is associated with adverse prognosis.^3^ Although this syndrome has been widely investigated, the molecular events driving SCM remain only partly clarified, and effective mechanism‐based therapies are still unavailable.^4^, ^5^

Cardiac fibroblasts have emerged as the key mediators of cardiac remodelling, particularly in the context of inflammation and fibrosis.^6^ These cells regulate extracellular matrix (ECM) turnover and, when abnormally activated, contribute to myocardial stiffening and contractile dysfunction.^7^ ADAMTS4 (A Disintegrin and Metalloproteinase with Thrombospondin Motifs 4) is a critical enzyme involved in ECM degradation, with potent aggrecanase activity.^8^ While ADAMTS4 has been extensively characterized in joint degeneration and pulmonary fibrosis^9^, ^10^—where it drives pathological remodelling through the potentiation of fibroblast activation^11^—its functional relevance in cardiac tissues, particularly in the context of sepsis‐induced cardiac injury, remains elusive.

Studies have shown that under lipopolysaccharide (LPS) stimulation, myocardial inflammatory cascades are triggered, promoting the release of pro‐inflammatory factors and inducing myocardial dysfunction such as oxidative stress and apoptosis.^12^ Concurrently, this process activates the transformation of cardiac fibroblasts into myofibroblasts, exacerbates extracellular matrix accumulation, and ultimately leads to myocardial fibrosis.^13^ Recent findings indicate that ADAMTS4 contributes to fibrotic progression by modulating ECM components such as aggrecan and versican, or by activating profibrotic signalling pathways in fibroblasts.^14^, ^15^ In pulmonary models, *Adamts4* expression is markedly elevated in activated fibroblasts and directly linked to impaired organ function.^16^ Given the shared mechanisms of fibrosis across organs, it is plausible that ADAMTS4 may similarly exacerbate cardiac fibrosis under septic conditions. However, no prior studies have addressed the role of ADAMTS4 in cardiac fibroblasts during sepsis.

Thrombospondin‑1 (TSP1, also known as THBS1) is a multifunctional matricellular glycoprotein rapidly upregulated in the extracellular matrix under inflammatory and stress conditions. As a pivotal upstream regulator of latent transforming growth factor‑β (TGF‑β) activation, full‑length TSP1 binds directly to the latency‑associated peptide (LAP) of the latent TGF‑β complex via its type I repeats, inducing conformational release of bioactive TGF‑β—a non‑proteolytic activation mechanism recognized as one of the most potent in vivo pathways.^17^ Activated TGF‑β subsequently drives cardiac fibroblast‑to‑myofibroblast transdifferentiation, α‐smooth muscle actin (α‐SMA) expression, and excessive collagen deposition, ultimately contributing to myocardial stiffness and contractile impairment. In cardiac pathologies, TSP1 exhibits context‑dependent duality: in pressure‑overload models, it exerts protective effects by preserving ECM integrity via TGF‑β signalling and matrix metalloproteinase (MMP) inhibition,^18^, ^19^ whereas in acute inflammatory milieus such as lipopolysaccharide (LPS)‑induced sepsis, TSP1 expression surges in both myocardial tissue and serum, where it amplifies cardiomyocyte oxidative injury, mitochondrial apoptosis and NF‑κB‑mediated inflammation, thereby exacerbating septic cardiomyopathy.^20^

Crucially, TSP1 is highly susceptible to limited proteolytic processing by multiple extracellular proteases, including members of the ADAMTS family as well as thrombin, neutrophil elastase, cathepsin G, and HtrA1. Such cleavage releases a bioactive N‐terminal heparin‐binding fragment (∼25 kDa) that, unlike the full‐length protein, promotes endothelial invasiveness and angiogenesis via MMP‐2/9 upregulation and TIMP‐2 suppression. In fibrosis, generation of this fragment may shift TSP1 from a matrix‐stabilizing protein toward a signal‐amplifying mediator, thereby promoting persistent TGF‐β/Smad and inflammatory signalling, fibroblast activation, and ECM deposition.^21^ Although ADAMTS1 and ADAMTS7 cleave TSP1 at distinct sites to generate larger fragments, the recurrent observation of a ∼25 kDa N‑terminal species across diverse protease systems underscores the functional significance of this cleavage event.^22^, ^23^, ^24^ Given that ADAMTS4 is a secreted metalloproteinase with established roles in ECM remodelling and has been reported to regulate TGF‐β‐related fibrotic responses through extracellular matrix substrates, we hypothesized that ADAMTS4 may contribute to septic cardiomyopathy by modifying TSP1‐dependent profibrotic signaling.^25^, ^26^ However, the role of ADAMTS4 in relation to TSP1 in septic cardiomyopathy remains unexplored. In this study, we aimed to investigate the function of *Adamts4* in septic cardiomyopathy, focusing on its fibrotic effects in cardiac fibroblasts. This novel mechanism sheds new light on the pathogenesis of SCM and suggests potential therapeutic targets for sepsis‐related cardiac dysfunction.

## 2 RESULTS

### 2.1 ADAMTS4 expression is significantly increased in LPS‐treated hearts and cardiac fibroblasts

**FIGURE 1:**
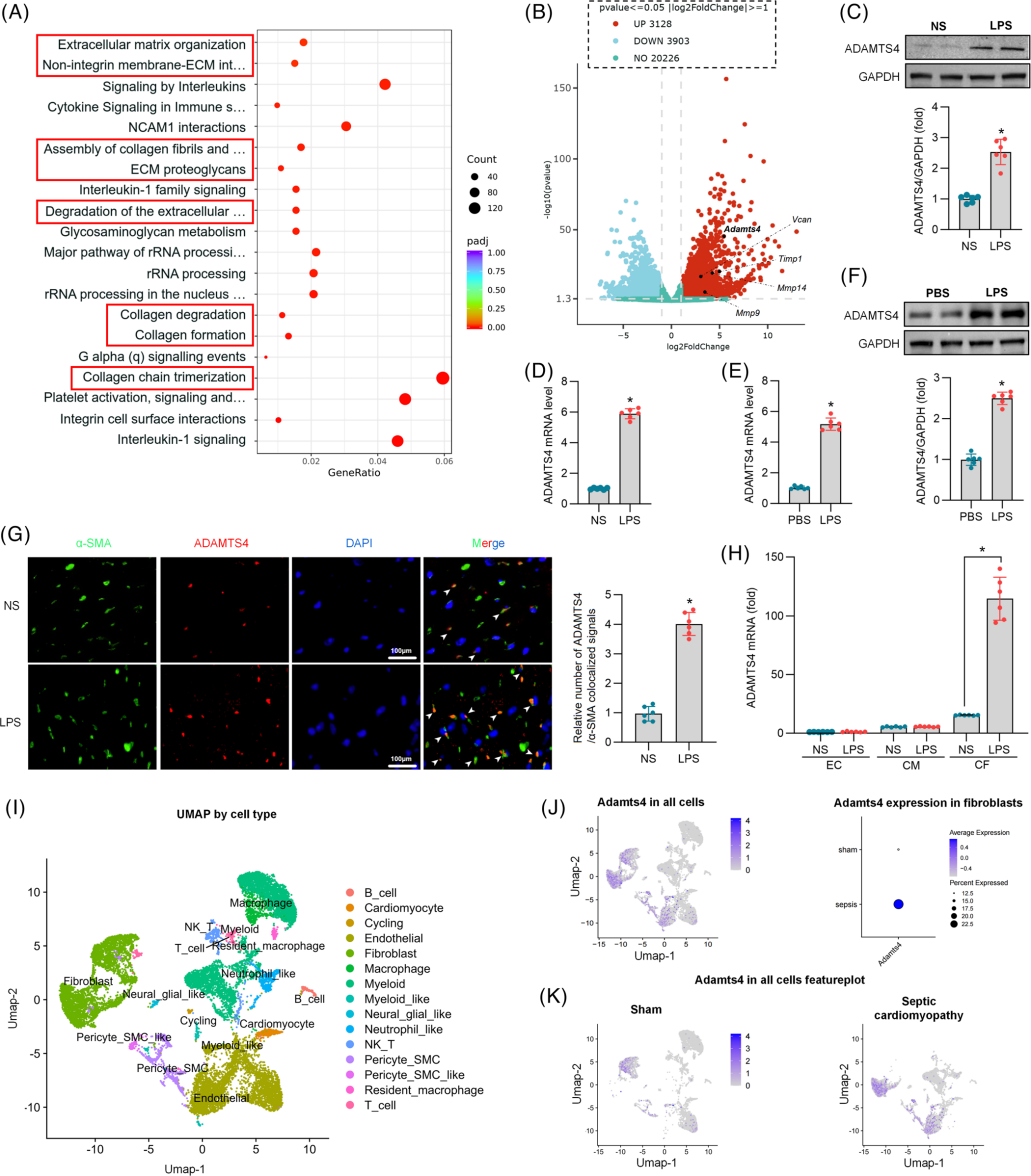
Adamts4 expression is increased in fibroblasts of septic mice. (A) Results of Reactome pathway enrichment analysis based on RNA‐seq data of mouse hearts after LPS stimulation (*n* = 6). (B) Volcano plot of differentially expressed genes in LPS‐induced septic cardiomyopathy. Matrisome‐related genes are highlighted, with representative ECM‐remodelling genes, including *Adamts4, Vcan, Timp1, Mmp14*, and *Mmp9*, specifically labelled. (C) Representative Western blot images and statistical analysis of Adamts4 protein expression in mouse hearts 24 h after LPS treatment (*n* = 6). (D) The level of *Adamts4* mRNA relative to NS group in mouse hearts 24 h after LPS treatment (*n* = 6). (E) Representative Western blot images and statistical analyses of the expression of ADAMTS4 protein in mouse fibroblasts following 24 h of LPS treatment (*n* = 6). (F) The relative mRNA level of *Adamts4* in mouse fibroblasts 24 h after LPS treatment (*n* = 6). (G) Representative immunofluorescence images showing α‐SMA, ADAMTS4 and DAPI staining in heart sections from NS‐ and LPS‐treated mice. Arrowheads indicate ADAMTS4‐positive signals overlapping with or spatially associated with α‐SMA‐positive regions. Quantification shows the relative number of ADAMTS4 signals associated with α‐SMA‐positive regions. DAPI was used as a nuclear counterstain to show tissue cellularity. (H) *Adamts4* mRNA levels in primary ECs, CMs, and CFs isolated from untreated wild‐type mouse hearts and subsequently stimulated with LPS in vitro for 24 h. (I) Reanalysis of the publicly available mouse cardiac single‐cell RNA‐seq dataset GSE207363. Uniform Manifold Approximation and Projection (UMAP) plot showing the major cardiac cell populations identified from the public scRNA‐seq dataset GSE207363. (J) Feature plot showing the distribution of *Adamts4* expression across different cardiac cell populations in the scRNA‐seq dataset. (K) Comparison of *Adamts4*‐positive cell distribution between sham and septic cardiomyopathy samples, showing that *Adamts4* expression is mainly enriched in fibroblast populations, with detectable changes in a subset of endothelial cells under septic conditions. Data are presented as mean ± SEM. Normality was assessed using the Shapiro–Wilk test. All tests were two‐sided. Two‐group comparisons were performed using an unpaired two‐tailed Student's *t*‐test. For comparisons involving more than two groups (Figure 1H), one‐way ANOVA with Tukey's post hoc test was applied following Levene's test for homogeneity of variance; Welch's ANOVA with Tamhane's T2 correction was used when variances were unequal. Non‐normally distributed data were analysed using the Mann–Whitney *U* test or Kruskal–Wallis test with Dunn's correction, as appropriate. Statistical significance was set at *p* < .05.

To investigate the molecular mechanisms underlying sepsis‐induced cardiac injury, we first established a murine model of acute sepsis via intraperitoneal administration of 15 mg/kg lipopolysaccharide (LPS) for 12 h, a condition selected to capture robust early transcriptional responses in the heart while minimizing interference from later‐stage terminal events associated with severe endotoxemia. To characterize the transcriptomic changes at the genome‐wide level, we performed RNA sequencing (RNA‐seq) on cardiac tissue samples isolated from mice with sepsis. Bioinformatic analyses, including Reactome pathway enrichment and volcano plot visualization, were conducted to characterize the biological processes and expression patterns of these DEGs (Figure 1A,B). These analyses revealed marked perturbations in multiple signalling pathways linked to collagen synthesis and extracellular matrix (ECM) turnover. Notably, when matrisome‐related genes were highlighted in the volcano plot, *Adamts4* was identified as a prominently upregulated secreted matrix protease after acute LPS stimulation, alongside other ECM‐associated genes such as *Vcan*, *Timp1*, *Mmp14* and *Mmp9*. Because *Adamts4* encodes an extracellular matrix‐associated protease and was consistently induced in subsequent validation experiments, we focused on *Adamts4* as a candidate mediator of LPS‐induced cardiac matrix remodelling.

To validate these transcriptomic findings, we challenged mice with 10 mg/kg LPS for 24 h and examined *Adamts4* expression in cardiac tissue. Relative to saline‐administered control mice, ADAMTS4 protein and mRNA were both increased in LPS‐treated hearts (Figure 1C,D). Although this validation experiment used a different LPS dose and time point from the RNA‐seq screening experiment, it was intended as an independent confirmation of Adamts4 induction rather than a direct quantitative comparison with the RNA‐seq condition. To strengthen the fibroblast‐specific response, we isolated cardiac fibroblasts from Adamts4^fl/fl^ mice and stimulated them with 10 µg/mL LPS. LPS treatment significantly increased ADAMTS4 expression at both the protein and transcript levels, consistent with our previous observations and supporting a prominent role for ADAMTS4 in cardiac fibroblast activation (Figure 1E,F). Immunofluorescence staining showed increased ADAMTS4 signal in LPS‐treated mouse hearts, with a higher number of ADAMTS4‐positive signals associated with α‐SMA‐positive fibroblast‐related regions (Figure 1G). Because ADAMTS4 is a secreted ECM‐associated metalloproteinase, part of the ADAMTS4‐positive signal may reflect extracellular or pericellular localization. To further characterize the cellular origin of Adamts4 under septic conditions, we purified primary endothelial cells (ECs), cardiomyocytes (CMs) and cardiac fibroblasts (CFs) from wild‐type mouse hearts and exposed each cell population to 10 µg/mL LPS. Quantitative real‐time PCR revealed that following acute in vitro LPS stimulation, Adamts4 mRNA was primarily upregulated in cardiac fibroblasts, whereas no appreciable alteration was observed in freshly isolated cardiomyocytes or endothelial cells (Figure 1H). Western blot analysis additionally validated strong induction of ADAMTS4 protein in LPS‐exposed cardiac tissues and isolated cardiac fibroblasts, whereas ADAMTS4 expression was barely detectable in cardiomyocytes and endothelial cells (Figure S1A–C).

To examine clinical relevance, we analysed public Gene Expression Omnibus (GEO) datasets and observed consistent *Adamts4* upregulation in septic cardiomyopathy across species, including human patients (GSE79962), LPS‐ and/or caecal ligation and puncture (CLP)‐challenged mice (GSE142615, GSE229925), and LPS‐treated rats (GSE266124). Notably, *Adamts4* expression was highest in the low‐LVEF subgroup, indicating a correlation between *Adamts4* upregulation and more severe cardiac dysfunction. This conserved expression pattern strongly corroborates our experimental results (Figure S1D–G).

To extend these findings at single‐cell resolution, we analysed the published scRNA‐seq dataset GSE207363. UMAP clustering identified major cardiac cell populations, including fibroblasts, endothelial cells, cardiomyocytes, macrophages and mural cells (Figure 1I). Cell‐type feature mapping showed that *Adamts4* expression was enriched primarily in fibroblast populations, while comparison between sham and septic cardiomyopathy samples further revealed an expanded *Adamts4*‐positive cell population in diseased hearts (Figure 1J,K). Notably, the scRNA‐seq data also suggested detectable changes in a subset of endothelial cells in vivo, indicating that endothelial *Adamts4* expression may be induced in a context‐dependent manner during septic cardiomyopathy.

Overall, these results show that *Adamts4* is markedly upregulated in the septic heart and that cardiac fibroblasts represent the major inducible source in our acute LPS‐based in vitro system, whereas single‐cell transcriptomic analysis of septic cardiomyopathy hearts suggests that additional cell populations, including endothelial subsets, may also contribute to Adamts4 expression in the complex in vivo microenvironment.

### 2.2 ADAMTS4 is a key regulator of cardiac function and myocardial fibrosis in mice

**FIGURE 2:**
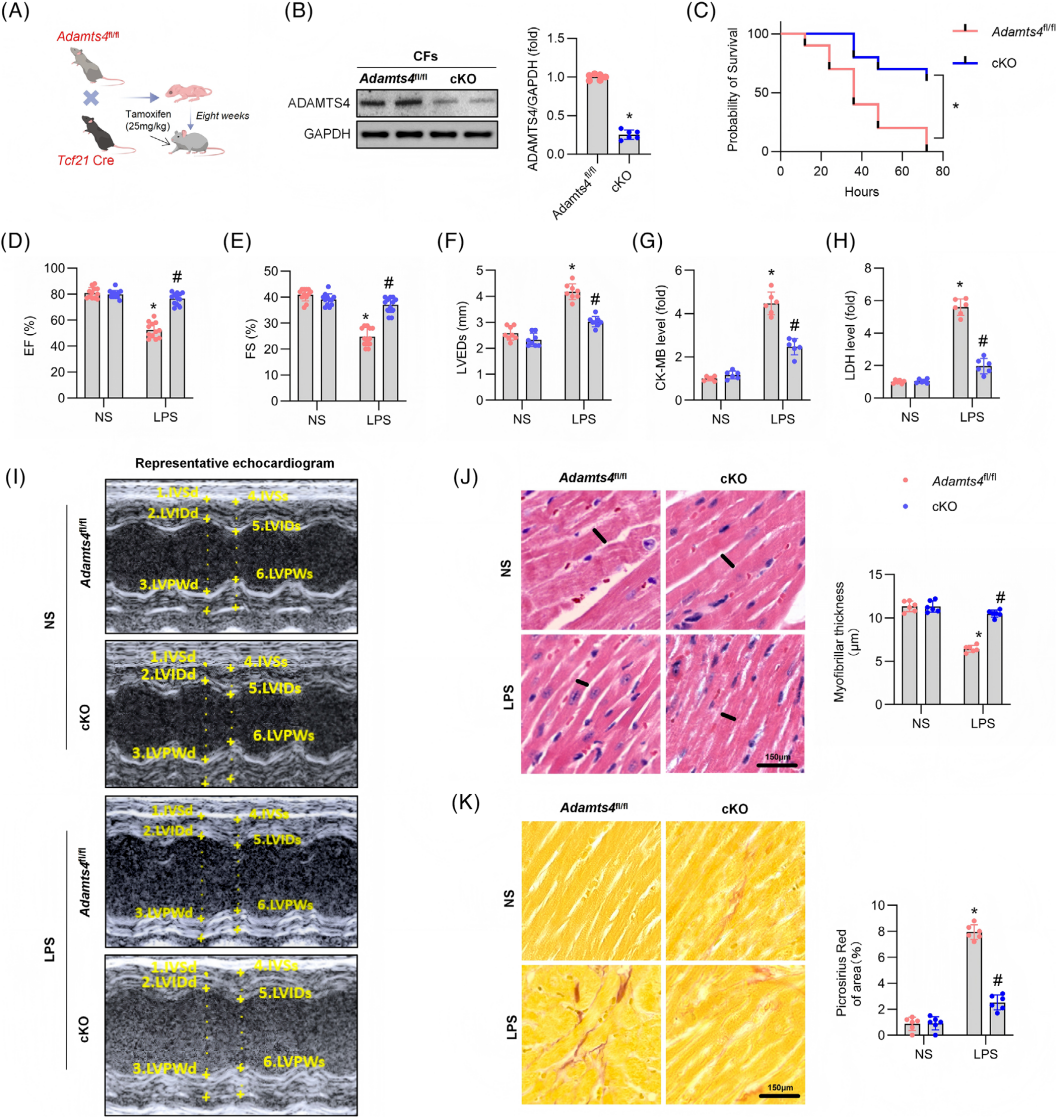
Tcf21‐lineage Adamts4‐deficient mice exhibit attenuated fibrosis and cardiac dysfunction in response to lipopolysaccharide (LPS) administration. (A) Tcf21‐lineage Adamts4‐deficient mice (Adamts4 cKO) were generated by crossing mice with conditional knockout alleles of *Adamts4* (Adamts4^fl/fl^) with Tcf21‐iCreERT2 mice. (B) Representative Western blot images and statistical analysis of Adamts4 protein expression in CFs isolated from Adamts4 cKO mice and littermate controls (*n* = 6). (C) Survival rate of mice challenged with LPS (15 mg/kg) (*n* = 12). (D and E) Changes in ejection fraction (EF) and fractional shortening (FS) in different groups of mice after LPS stimulation (*n* = 12). (F) Changes in left ventricular end‐systolic diameter (LVESD) after LPS stimulation in different groups of mice (*n* = 8). (G and H) Changes in levels of LDH and CK‐MB in mouse serum after LPS stimulation in different groups of mice (*n* = 6). (I) Representative echocardiograms of different groups of mice. (J) H&E staining. The black line indicates the thickness of myofibrils (*n *= 6), and myofibrils become thinner after LPS stimulation. (K) PSR staining. The area of cardiac tissue fibrosis in Adamts4 cKO mice decreases after LPS stimulation (*n* = 6). Data are presented as mean ± SEM. Normality was assessed using the Shapiro–Wilk test. All tests were two‐sided. For two‐group comparisons (Figure 2B), an unpaired two‐tailed Student's *t*‐test was used. For multiple comparisons (Figure 2D–K), one‐way ANOVA with Tukey's post hoc test was applied following confirmation of variance homogeneity by Levene's test; Welch's ANOVA with Tamhane's T2 correction was used when variances were unequal. Survival analysis (Figure 2C) was performed using the Kaplan–Meier method with the log‐rank (Mantel–Cox) test. Non‐parametric alternatives (Mann–Whitney *U* test or Kruskal–Wallis test with Dunn's correction) were employed when data were non‐normally distributed. Analyses were performed using GraphPad Prism 10. A *p* value < .05 was considered statistically significant. * *p* < .05 versus littermate Adamts4^fl/fl^ controls; # *p* < .05 compared with the LPS‐stimulated Adamts4 cKO group.

To define the role of Adamts4 in sepsis‐induced fibrosis, we selected three canonical fibroblast markers—Transcription Factor 21 (*Tcf21*), Collagen type I (*Col1a2*), and Periostin (*Postn*)—and performed six independent animal experiments to ensure robust evidence. We first employed the tamoxifen‐inducible Tcf21‐iCreERT2 driver to manipulate Adamts4 expression in Tcf21‐lineage fibroblasts.^27^ Adamts4^fl/fl^ mice were crossed with Tcf21‐iCreERT2 mice to generate Tcf21^iCreERT2/+^; Adamts4^fl/fl^ mice, hereafter referred to as Adamts4 cKO mice. Adult mice received tamoxifen intraperitoneally at 25 mg/kg/day for five consecutive days to induce iCreERT2‐mediated recombination in Tcf21‐lineage cells (Figure 2A). Adamts4 cKO mice exhibited normal viability and fertility, with no apparent gross abnormalities. Following a 2‐week tamoxifen washout period, freshly isolated cardiac fibroblasts (CFs) from cKO mice showed reduced ADAMTS4 protein levels compared to those isolated from littermate Adamts4^fl/fl^ mice, confirming successful model establishment (Figure 2B).

After high‐dose LPS exposure (15 mg/kg), Adamts4 cKO mice showed improved survival compared with their littermate controls. However, high‐dose LPS‐induced mortality reflects systemic endotoxaemic stress rather than cardiac dysfunction alone. Thus, the survival benefit observed after fibroblast‐lineage Adamts4 deletion does not establish a direct causal relationship between improved cardiac function and reduced mortality (Figure 2C). To assess LPS‐associated cardiac dysfunction and pathological fibrotic remodelling under a repeated inflammatory stimulus, we administered LPS at 5 mg/kg by intraperitoneal injection once weekly for two consecutive weeks, and tissues were harvested 7 days after the second injection. Echocardiographic analysis revealed that LPS treatment significantly increased left ventricular end‐systolic diameter (LVESD) and decreased ejection fraction (EF) and fractional shortening (FS), while elevating serum levels of myocardial injury markers creatine kinase‐MB (CK‐MB) and lactate dehydrogenase (LDH) (Figure 2D–H). Representative echocardiographic images are shown in Figure 2I. Notably, these pathological changes were attenuated in Adamts4 cKO mice, indicating that Tcf21‐lineage fibroblast Adamts4 deletion mitigates LPS‐induced cardiac dysfunction and injury. Histological analysis further confirmed these findings, demonstrating that LPS‐induced loss of myofibril thickness was alleviated in Adamts4 cKO mice (Figure 2J). Picrosirius red (PSR) staining revealed pronounced cardiac fibrosis in LPS‐treated Adamts4^fl/fl^ mice, which was markedly reduced in Adamts4 cKO hearts (Figure 2K). Collectively, these data suggest that fibroblast‐lineage Adamts4 contributes critically to LPS‐induced myocardial fibrosis and injury.

**FIGURE 3:**
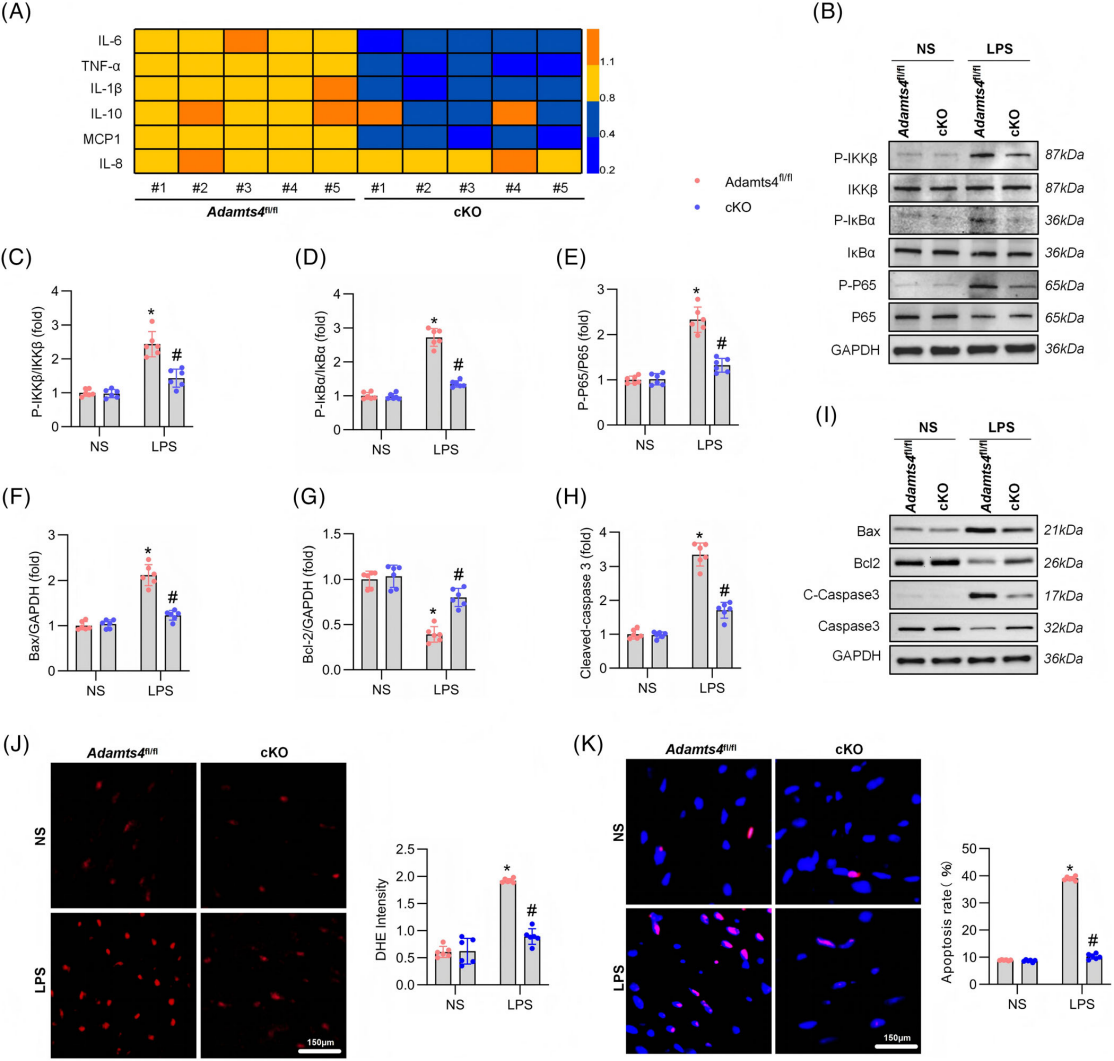
Tcf21‐lineage Adamts4‐deficient mice display attenuated myocardial inflammation in response to lipopolysaccharide (LPS) administration. (A) The expression levels of cytokines such as *IL‐6, TNF‐α, IL‐1β, IL‐10, MCP1* and *IL‐8* in samples from Adamts4^fl/fl^ mice (control group) and Adamts4 cKO mice were detected, and the right‐side scale shows the relative expression fold (*n* = 5). (B–E) The protein expression of inflammation‐related indicators IKKβ, phosphorylated IKKβ (p‐IKKβ), IκBα, phosphorylated IκBα (p‐IκBα), p65, and phosphorylated p65 (p‐p65) was detected (*n* = 6). (F–I) The protein expression levels of apoptosis‐related indicators Bax, Bcl‐2, Caspase‐3 and Cleaved‐Caspase‐3 were detected (*n* = 6). (J) Representative images of DHE distribution detected by immunofluorescence, and the DHE fluorescence intensity was counted (*n* = 6). (K) The TUNEL method was used to detect the apoptosis of cells in myocardial tissue sections of mice in different groups, and the cell apoptosis rate was calculated (*n* = 6). Data are presented as mean ± SEM. Normality was assessed using the Shapiro–Wilk test. All tests were two‐sided. For two‐group comparisons (Figure 3B), an unpaired two‐tailed Student's *t*‐test was used. For multiple comparisons (Figure 3A,C–K), one‐way ANOVA with Tukey's post hoc test was applied following confirmation of variance homogeneity by Levene's test; where variances were unequal, Welch's ANOVA with Tamhane's T2 correction was used. Non‐parametric tests (Mann–Whitney *U* test or Kruskal–Wallis test with Dunn's correction) were employed when data deviated from normality. Statistical significance was set at P < 0.05. * *p* < .05 versus littermate Adamts4^fl/fl^ controls; # *p* < .05 compared with the LPS‐stimulated Adamts4 cKO group.

Next, we assessed myocardial inflammation using quantitative real‐time PCR (qRT‐PCR). In Adamts4 cKO mice, the expression of inflammatory cytokines, including *IL‐6, TNF‑α, IL‑1β, IL‑10* and *MCP1*, was significantly reduced, whereas *IL‐8* levels remained largely unchanged (Figure 3A). Western blot analysis revealed that LPS stimulation robustly increased the phosphorylation of IKKβ, IκBα and NF‐κB p65 in Adamts4^fl/fl^ mice. These phosphorylation events were substantially diminished in Adamts4 cKO mice following LPS challenge, while total protein levels were unaffected. These data indicate that Tcf21‐lineage Adamts4 deletion mitigates LPS‐induced activation of the canonical NF‐κB signalling pathway in the heart (Figure 3B–E). We therefore conducted further experiments to evaluate the effect of ADAMTS4 on superoxide production. Immunofluorescence staining demonstrated that Tcf21‐lineage Adamts4 deletion significantly reduced LPS‐induced intracellular superoxide generation in the heart (Figure 3J).

Apoptotic cell death in myocardial tissue is associated with LPS‐induced cardiomyopathy.^12^, ^28^ Adamts4 deletion reduced the expression of the pro‐apoptotic protein Bax and increased the expression of the anti‐apoptotic protein Bcl‐2. In addition, Adamts4 deficiency increased total Caspase‐3 levels but markedly decreased cleaved Caspase‐3 levels (Figure 3F–I). Further analysis revealed that Compared with saline‐injected animals, LPS‐treated animals showed an increased number of TUNEL‐positive apoptotic cells in myocardial sections, whereas Adamts4 cKO mice injected with LPS showed a lower apoptotic burden (Figure 3K). Because TUNEL staining alone does not define the identity of apoptotic cells, these data were interpreted as reflecting apoptotic cell death in myocardial tissue rather than cardiomyocyte‐specific apoptosis. Collectively, these findings indicate that Adamts4 deletion alleviates LPS‐induced apoptosis‐related myocardial injury.

Because the Tcf21‐ and Postn‐lineage models were used as the principal fibroblast‐lineage and injury‐activated myofibroblast models in the main figures, the Col1a2‐CreER model was included as a complementary validation to assess Adamts4 function in a broader collagen‐producing fibroblast compartment. Adamts4^fl/fl^ mice were crossed with Col1a2‐CreER mice to obtain Col1a2‐lineage *Adamts4*‐deficient mice, hereafter referred to as Adamts4 cfKO mice (Figure S2A). This model was used to determine whether deletion of *Adamts4* in a broader fibroblast population could also protect against LPS‐induced cardiac injury. Adamts4 cfKO mice were born at the expected frequency and showed no apparent abnormalities in viability, fertility or gross phenotype. After LPS challenge, cardiac fibroblasts isolated from Adamts4 cfKO mice displayed markedly lower ADAMTS4 protein expression than those from littermate Adamts4^fl/fl^ controls, verifying efficient establishment of the cfKO model (Figure S2B).

We first assessed survival rates and cardiac function. High‐dose LPS (15 mg/kg) challenge resulted in significantly improved survival in *Adamts4* cfKO mice compared to Adamts4^fl/fl^ littermates (Figure S2C). Following intraperitoneal injection of LPS at 5 mg/kg once weekly for two consecutive weeks, Adamts4 cfKO mice exhibited improved cardiac function, with ejection fraction and fractional shortening approaching normal levels and myocardial injury markers CK‐MB and LDH significantly reduced (Figure S2D–G). Histological staining and immunofluorescence revealed that Col1a2‐lineage Adamts4 deletion significantly reduced LPS‐induced intracellular superoxide production (Figure S2H). TUNEL‐positive apoptotic cells in myocardial sections were reduced in Adamts4 cfKO mice and remained at levels comparable to those in saline‐injected controls (Figure S2I). In addition, consistent with findings in Adamts4 cKO mice, LPS‐induced fibrosis was markedly ameliorated in Adamts4 cfKO hearts (Figure S2J).

**FIGURE 4:**
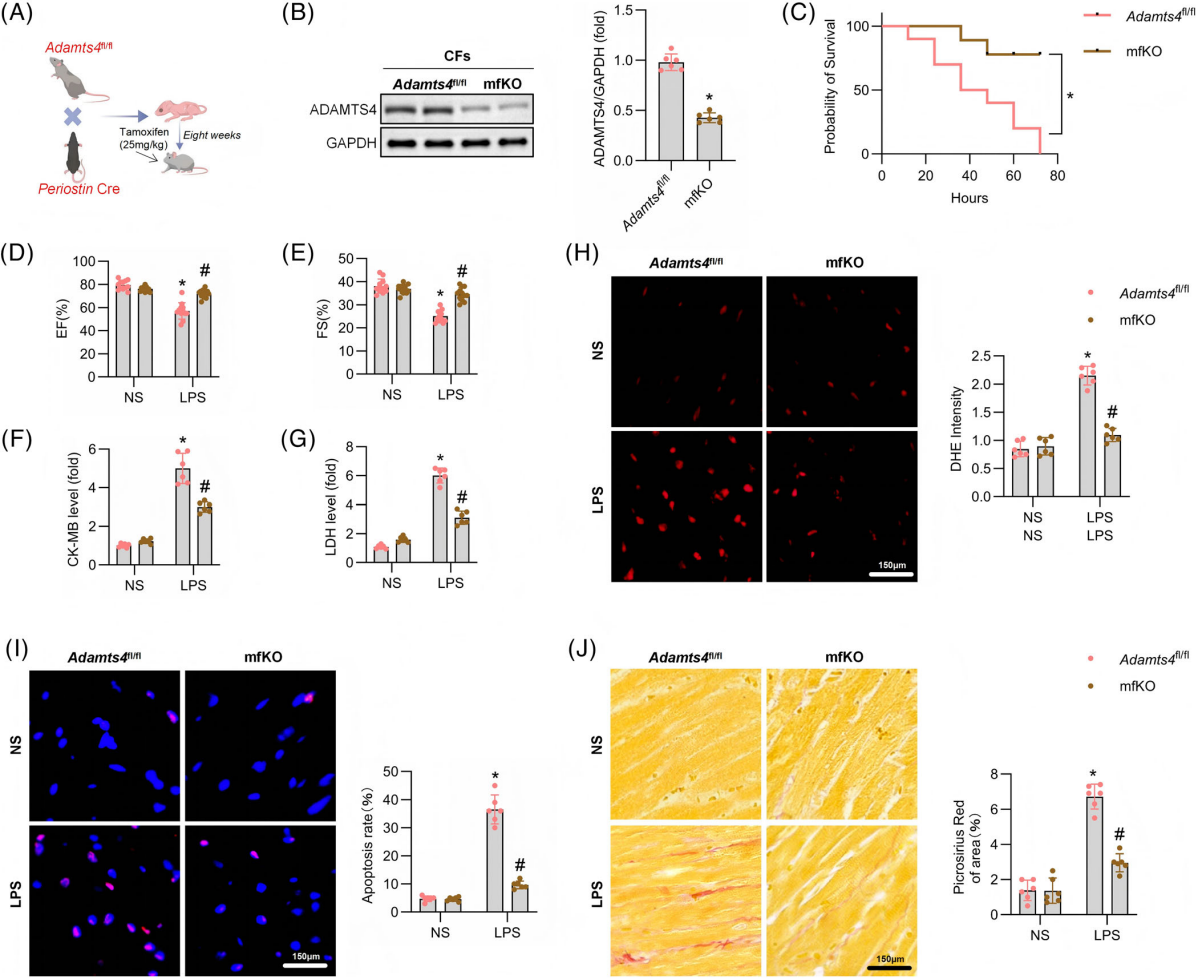
Postn‐lineage Adamts4‐deficient mice exhibit attenuated LPS‐induced cardiac dysfunction and myocardial fibrosis. (A and B) By crossing Adamts4 knockout mice (Adamts4^fl/fl^) with Postn‐CreER mice, we successfully developed Postn‐lineage Adamts4‐deficient mice. The figure displays representative Western blot images and statistical analysis results of Adamts4 protein expression in cardiac fibroblasts (CFs) isolated from Adamts4 mfKO mice and littermate Adamts4^fl/fl^ controls (*n* = 6). (C) Survival curves of mice challenged with LPS (15 mg/kg) (*n* = 12). (D and E) Dynamic changes in ejection fraction (EF) and fractional shortening (FS) in different groups of mice after LPS stimulation (*n* = 12). (F and G) Changes in serum levels of lactate dehydrogenase (LDH) and creatine kinase‐MB (CK‐MB) in different groups of mice after LPS stimulation (*n* = 6). (H) Representative immunofluorescence images showing DHE distribution and quantification of DHE fluorescence intensity (*n* = 6). (I) TUNEL staining and quantification of apoptotic cells in myocardial sections from different groups of mice (*n* = 6). (J) PSR staining showing reduced cardiac fibrosis area in Adamts4 mfKO mice after LPS stimulation (*n* = 6). Data are presented as mean ± SEM. Normality was assessed using the Shapiro–Wilk test. All tests were two‐sided. For two‐group comparisons (Figure 4B), an unpaired two‐tailed Student's *t*‐test was used. For multiple comparisons (Figure 4D–J), one‐way ANOVA with Tukey's post‐hoc test was applied following Levene's test for homogeneity of variance; Welch's ANOVA with Tamhane's T2 correction was used when variances were unequal. Survival analysis (Figure 4C) was performed using the Kaplan–Meier method with the log‐rank (Mantel–Cox) test. Non‐normally distributed data were analysed using the Mann–Whitney *U* test or Kruskal–Wallis test with Dunn's correction. Statistical significance was set at *p* < .05. * *p *< .05 versus littermate Adamts4^fl/fl^ controls; # *p* < .05 compared with the LPS‐stimulated Adamts4 mfKO group.

To target injury‐activated myofibroblast‐lineage cells during fibrosis, we generated Postn‐lineage Adamts4‐deficient mice, hereafter referred to as Adamts4 mfKO mice, using a similar conditional recombination strategy (Figure 4A). Given that Periostin is minimally expressed in healthy hearts and serves as a specific marker for activated myofibroblasts post‐injury,^29^ Postn‐CreER was employed to preferentially delete Adamts4 in this injury‐activated fibroblast/myofibroblast‐lineage compartment. Cardiac fibroblasts (CFs) isolated from LPS‐stimulated *Adamts4* mfKO mice exhibited significantly reduced *Adamts4* expression compared to littermate controls, confirming successful model establishment (Figure 4B).

Following high‐dose LPS administration (15 mg/kg), Adamts4 mfKO mice displayed a higher survival probability than their Adamts4^fl/fl^ littermate controls (Figure 4C). In line with the results obtained from the cKO and cfKO models, loss of Adamts4 in Postn‐lineage myofibroblasts improved cardiac performance, as reflected by preserved EF and FS, together with lower circulating CK‐MB and LDH levels (Figure 4D–G). Immunofluorescence staining further showed reduced intracellular superoxide accumulation and fewer TUNEL‐positive apoptotic cells in myocardial sections after Postn‐lineage Adamts4 deletion (Figure 4H,I). LPS‐induced collagen deposition was also substantially decreased in Adamts4 mfKO hearts (Figure 4J), reinforcing the protective effect of Adamts4 ablation across multiple fibroblast‐lineage compartments.

Because ADAMTS4 is a secreted, extracellular matrix‐associated protease that may be released into the circulation under inflammatory conditions, we further measured ADAMTS4 levels in serum and cardiac tissue. ELISA analysis showed that LPS stimulation significantly increased serum ADAMTS4 concentrations compared with the normal saline (NS) group (Figure S1H). Cardiac tissue ADAMTS4 content was also significantly increased following LPS stimulation (Figure S1I). Although the absolute values cannot be directly compared because of differences in measurement units and normalization methods, the relative increase in cardiac ADAMTS4 was more pronounced than that observed in serum. These findings indicate that systemic LPS challenge increases ADAMTS4 levels in both cardiac tissue and the circulation, with a more prominent change occurring in the heart. However, these data do not yet rule out a potential contribution of Adamts4 from extracardiac tissues.

After establishing the protective effects of Adamts4 deletion across three fibroblast‐lineage models, we further generated cardiomyocyte‐specific Adamts4 knockout mice (Adamts4 cmKO) using α‐MHC‐MerCreMerα‐MHC‐Cre and Tie2‐lineage Adamts4‐deficient mice (Adamts4 ecKO) using Tie2‐iCreERT2 (Figures S3A and S4A). Although cardiac fibroblasts were identified as the major inducible source of Adamts4 after LPS stimulation, these additional models were generated to exclude potential contributions from other major cardiac cell lineages. Thus, the cmKO and ecKO mice served as negative lineage controls and helped further define the fibroblast‐lineage‐dependent role of Adamts4 in LPS‐induced cardiac injury and remodelling. Primary cardiomyocytes (CMs) isolated from Adamts4 cmKO mice and primary endothelial cells (ECs) from Adamts4 ecKO mice showed significantly reduced Adamts4 protein expression compared to littermate Adamts4^fl/fl^ controls via Western blotting, confirming successful model establishment (Figures S3B and S4B).

Notably, high‐dose LPS challenge did not alter the mortality of Adamts4 cmKO or ecKO mice relative to littermate controls (Figures S3C and S4C). Neither cardiac function parameters (EF, FS) nor myocardial injury markers (CK‐MB, LDH) differed significantly between knockout mice and controls after LPS stimulation (Figures S3D–G and S4D–G). Histological analysis further revealed no significant difference in myocardial fibrosis area between LPS‐treated knockout mice and controls (Figures S3H and S4H). These results demonstrate that Adamts4 mediates LPS‐induced cardiac fibrosis and dysfunction in a cell‐specific manner, acting primarily through fibroblasts rather than cardiomyocytes or endothelial cells.

**FIGURE 5:**
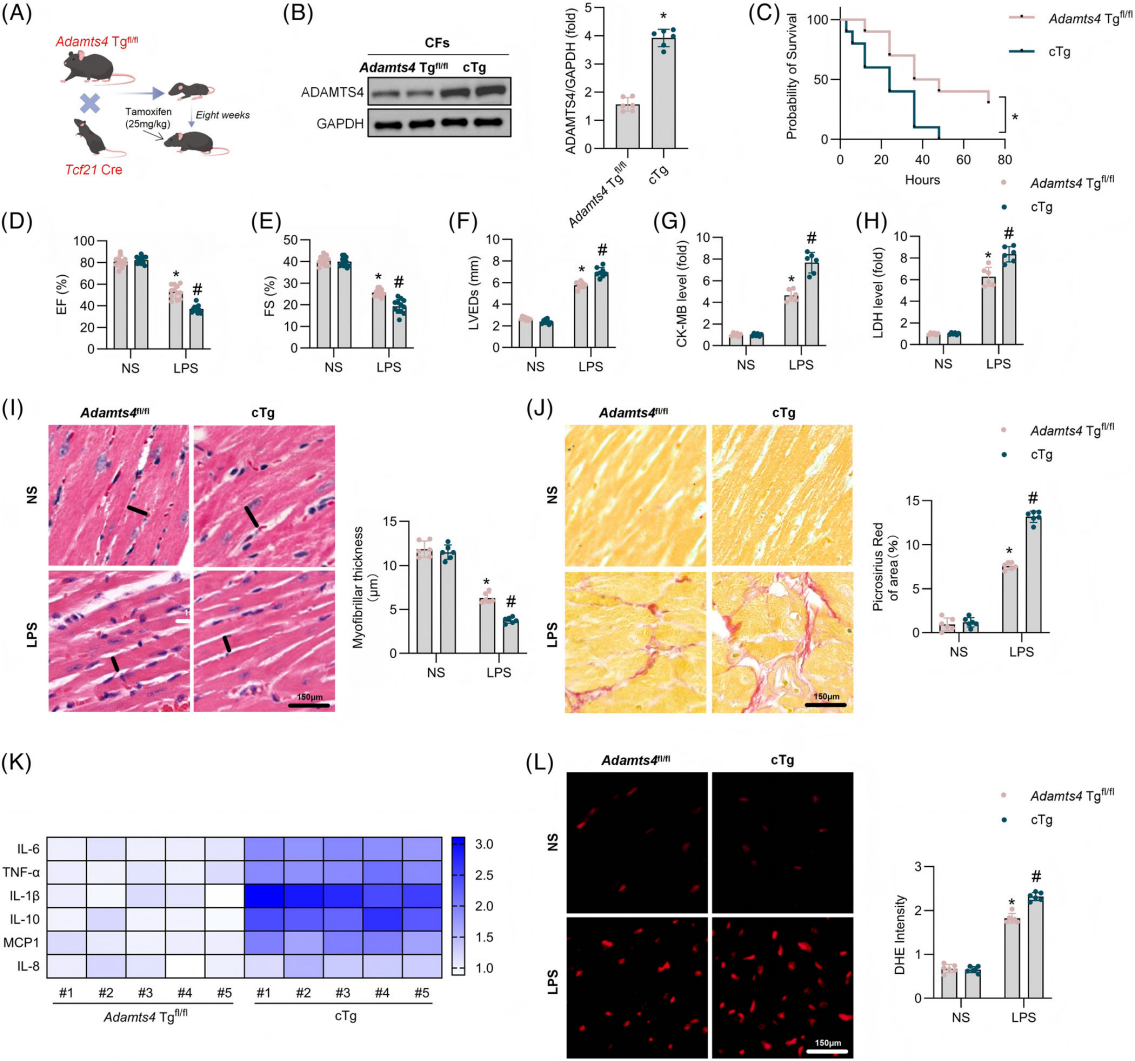
Tcf21‐lineage fibroblast *Adamts4* overexpression exacerbates lipopolysaccharide (LPS)‐induced myocardial fibrosis and cardiac dysfunction. (A and B) Conditional Tcf21‐lineage fibroblast *Adamts4* overexpression mice (Adamts4 cTg) were generated by crossing Adamts4 Tg^fl/fl^ conditional transgenic with Tcf21‐iCreERT2 mice. Representative Western blot images and statistical analysis of Adamts4 protein expression in cardiac fibroblasts (CFs) isolated from Adamts4 cTg mice and littermate controls (*n* = 6). (C) Survival rate of mice challenged with LPS (15 mg/kg) (*n* = 12). (D and E) Changes in ejection fraction (EF) and fractional shortening (FS) in different groups of mice after LPS stimulation (*n* = 12). (F) Changes in left ventricular end‐systolic diameter (LVESD) in different groups of mice after LPS stimulation (*n* = 8). (G and H) Changes in levels of LDH and CK‐MB in mouse serum in different groups of mice after LPS stimulation (*n* = 6). (I) H&E staining: Black lines indicate the thickness of myofibrils (*n* = 6). Myofibrils in *Adamts4* cTg mice were thinner after LPS stimulation. (J) PSR staining: After LPS stimulation, the fibrosis area of cardiac tissue in Adamts4 cTg mice increased (*n* = 6). (K) The expression levels of cytokines such as *IL‐6, TNF‐α, IL‐1β, IL‐10, MCP1* and *IL‐8* in samples from Adamts4 Tg^fl/fl^ mice (control group) and Tcf21‐lineage fibroblast *Adamts4* overexpression (cTg) mice were detected, and the right‐side scale shows the relative expression fold (*n* = 5). (L) Representative immunofluorescence images showing DHE distribution and quantification of DHE fluorescence intensity in different groups of mice (*n* = 6). Data are presented as mean ± SEM. Normality was assessed using the Shapiro–Wilk test. All tests were two‐sided. For two‐group comparisons (Figure 5B), an unpaired two‐tailed Student's *t*‐test was used. For multiple comparisons (Figure 5D–L), one‐way ANOVA with Tukey's post‐hoc test was applied following Levene's test for homogeneity of variance; Welch's ANOVA with Tamhane's T2 correction was used when variances were unequal. Survival analysis (Figure 5C) was performed using the Kaplan–Meier method with the log‐rank (Mantel–Cox) test. Non‐normally distributed data were analysed using the Mann–Whitney *U* test or Kruskal–Wallis test with Dunn's correction. Statistical significance was set at *p* < .05. *p < .05 versus littermate Adamts4 Tg^fl/fl^ controls; # *p* < .05 compared with the LPS‐stimulated Adamts4 cTg group.

Following the loss‐of‐function studies, we performed gain‐of‐function experiments to determine whether Tcf21‐lineage Adamts4 overexpression aggravates LPS‐induced cardiac injury and fibrotic remodelling. Conditional Adamts4 transgenic mice (Adamts4 Tg^fl/fl^) were crossed with tamoxifen‐inducible Tcf21‐iCreERT2 mice. Following tamoxifen administration, iCreERT2‐mediated recombination generated Tcf21^iCreERT2/+^; Adamts4 Tg^fl/fl^ mice, hereafter referred to as Adamts4 cTg mice (Figure 5A). Western blotting confirmed that cardiac fibroblasts (CFs) from Adamts4 cTg mice expressed markedly higher levels of ADAMTS4 than CFs from littermate Adamts4 Tg^fl/fl^ controls, validating the overexpression model (Figure 5B). Under high‐dose LPS exposure, Adamts4 cTg mice showed poorer survival than their littermate controls (Figure 5C). Following repeated low‐dose LPS stimulation, Adamts4 cTg mice developed more severe cardiac dysfunction, as indicated by decreased EF and FS together with an enlarged LVESD (Figure 5D–F). Histological analysis further showed greater myofibrillar thinning in LPS‐treated Adamts4 cTg hearts (Figure 5I). Consistently, circulating CK‐MB and LDH levels were higher in the LPS‐treated cTg group, accompanied by an expanded fibrotic area in the myocardium (Figure 5G–H,J). These results indicate that fibroblast‐lineage *Adamts4* overexpression aggravates LPS‐induced cardiac injury and fibrotic remodelling.

Quantitative real‐time PCR (qRT‐PCR) indicated that Adamts4 cTg mice exhibited elevated expression of pro‐inflammatory cytokines, including *IL‐6, TNF‑α, IL‐1β, IL‐10* and *MCP1*, whereas *IL‐8* levels remained largely unchanged (Figure 5K). As revealed by Western blotting, relative to treatment with LPS alone, Adamts4 cTg mice showed significantly increased cleaved Caspase‐3 and Bax abundance, together with reduced Bcl‐2 abundance (Figure S5A–D), which was in stark contrast to the changes in Adamts4 cKO mice. Histological validation demonstrated that Adamts4 overexpression increased myocardial reactive oxygen species (ROS) production (Figure 5L) and aggravated apoptotic cell death in myocardial tissue (Figure S5E) under LPS stimulation.

### 2.3 ADAMTS4 promotes cardiac fibroblast activation through direct interaction with and cleavage of TSP1

To complement the in vitro experimental evidence, we performed a series of cellular assays in mouse cardiac fibroblasts (CFs), primarily using an shRNA‐mediated knockdown strategy. *Adamts4* expression in mouse‐derived CFs was efficiently reduced by short hairpin RNA targeting *Adamts4* (*shAdamts4*), as confirmed by Western blotting (Figure S6A). In parallel, we established an *Adamts4* overexpression system by transducing CFs with a lentiviral vector encoding the full‐length coding sequence of mouse *Adamts4*. This approach resulted in robust *Adamts4* overexpression (*oeAdamts4*), which was likewise verified by Western blot analysis (Figure S6B). We first examined the effects of *Adamts4* manipulation on inflammatory responses and cell viability. Under LPS stimulation, *shAdamts4* significantly attenuated the induction of the pro‐inflammatory cytokines IL‐1β and TNF‐α and markedly improved CF viability at 24 h (Figure S7A,B,G). In contrast, Adamts4 overexpression enhanced LPS‐induced IL‐1β and TNF‐α expression and significantly decreased CF viability at 24 h (Figure S7C,D,H). These findings indicate that ADAMTS4 exacerbates inflammatory injury and compromises fibroblast viability in response to LPS challenge.

**FIGURE 6:**
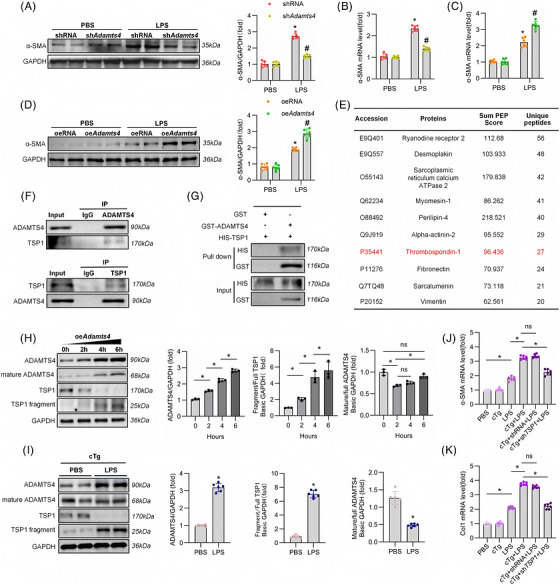
ADAMTS4 promotes cardiac fibroblast activation through direct interaction with and cleavage of TSP1. (A) Mouse cardiac fibroblasts (CFs) were transfected with shRNA targeting Adamts4 (*shAdamts4*) or control shRNA and stimulated with LPS. Protein expression of α‐SMA was assessed by Western blotting and quantified relative to GAPDH. (B) CFs with *Adamts4* knockdown were stimulated with LPS, and α‐SMA mRNA levels were measured by qPCR. (C) CFs were infected with lentivirus encoding full‐length Adamts4 (*oeAdamts4*) or control vector and stimulated with LPS. Protein expression of α‐SMA was analysed by Western blotting and quantified relative to GAPDH. (D) CFs overexpressing Adamts4 were stimulated with LPS, and α‐SMA mRNA levels were measured by qPCR. (E) Co‐immunoprecipitation (Co‐IP) combined with LC‐MS analysis was performed using heart tissue lysates from LPS‐treated mice. Representative high‐confidence candidate proteins are listed, with thrombospondin‐1 (TSP1) identified as a potential interacting partner of ADAMTS4. (F) Reciprocal Co‐IP assays were performed in heart tissue lysates using antibodies against ADAMTS4 or TSP1, followed by immunoblotting to validate the interaction between ADAMTS4 and TSP1. (G) GST pull‐down assay was conducted to determine whether ADAMTS4 directly binds to TSP1. GST‐tagged ADAMTS4 and HIS‐tagged TSP1 were used, and bound proteins were detected by immunoblotting. (H) Time‐course analysis of ADAMTS4‐mediated TSP1 processing in CFs overexpressing *Adamts4* following LPS stimulation. Cells and conditioned supernatants were collected at the indicated time points. Full‐length intracellular proteins were normalized to GAPDH, whereas secreted TSP1‐derived fragments in conditioned media were normalized to total membrane protein staining. (I) CFs isolated from Adamts4 cTg mice and their littermate controls were stimulated with LPS. Protein levels of ADAMTS4, full‐length TSP1, and TSP1 fragments were analysed by Western blotting and quantified. (J) CFs were subjected to *Adamts4* overexpression with or without *Tsp1* knockdown and stimulated with LPS. *α‐SMA* mRNA levels were measured by qPCR. (K) CFs were subjected to Adamts4 overexpression with or without Tsp1 knockdown and stimulated with LPS. Col1a1 mRNA levels were measured by qPCR. Data are presented as mean ± SEM. Normality and homogeneity of variance were assessed using Shapiro‐Wilk and Levene's tests, respectively. Two‐group comparisons were made using unpaired two‐tailed Student's *t*‐test or Mann–Whitney U test, as appropriate. Multiple‐group comparisons were performed using one‐way ANOVA with Tukey's post hoc test (equal variances) or Tamhane's T2 test (unequal variances); non‐normally distributed data were analysed via Kruskal–Wallis test with Dunn's post‐hoc correction. All tests were two‐sided, with *p* < .05 considered significant. * *p* < .05 versus respective control group; # *p* < .05 versus the corresponding LPS‐treated control.

We next focused on fibrosis‐related endpoints. Under LPS stimulation, Adamts4 silencing markedly suppressed the LPS‐induced increase in α‐SMA protein abundance, as shown by Western blotting (Figure 6A), and this inhibitory effect was also observed at the mRNA level, where α‐SMA expression was significantly reduced (Figure 6B). Conversely, *Adamts4* overexpression significantly increased both α‐SMA mRNA and protein levels in LPS‐treated CFs (Figure 6C,D). Collectively, these results identify ADAMTS4 as a key regulator of fibroblast activation and support its role in promoting the transition of fibroblasts toward a myofibroblast‐like phenotype under septic stress.

To identify the substrates through which ADAMTS4 mediates fibroblast activation, we performed co‐immunoprecipitation (Co‐IP) coupled with LC‐MS analysis using heart tissues from LPS‐stimulated Adamts4^fl/fl^ mice. Following differential protein screening, KEGG pathway enrichment analysis revealed significant enrichment in categories associated with cardiovascular disease (Figure S7I). In total, we identified 570 candidate interacting proteins, including 73 proteins associated with myocardial disease, from which the top 10 candidates were prioritized according to peptide confidence scores (Figure 6E). Among these candidates, TSP1 (P35441) was detected with high confidence, with 27 unique peptides and a peptide score of 96.436, supporting its robust enrichment in the ADAMTS4 immunoprecipitation complex. Importantly, α‐actinin‐2, one of the other top‐ranked candidates, is a sarcomeric Z‐disc protein and is therefore less readily accessible as a direct extracellular substrate of the secreted metalloproteinase ADAMTS4,^30^ the same applies to ryanodine receptor 2 and desmoplakin. By contrast, TSP1 is a well‐characterized extracellular matrix glycoprotein that co‐localizes with ADAMTS4 in the extracellular space, making it a biologically plausible substrate. Furthermore, prior evidence has shown that TSP1 can be cleaved by ADAMTS family proteases, further supporting its candidacy.^23^ Therefore, we considered TSP1 to be a more specific and biologically relevant substrate for mechanistic investigation in our model.

We then tested whether ADAMTS4 physically interacts with TSP1. Reciprocal Co‐IP assays confirmed an interaction between the two proteins in heart tissue lysates (Figure 6F). To further determine whether this interaction is direct, we performed a GST pull‐down assay using GST‐tagged ADAMTS4. Consistent with the Co‐IP results, ADAMTS4 was detected in complex with TSP1, demonstrating direct binding between the two proteins (Figure 6G).

Having established this interaction, we next investigated whether ADAMTS4 regulates TSP1 processing. To this end, we performed a time‐course experiment in *oeAdamts4* CFs stimulated with LPS. Cells and conditioned supernatants were collected at 0, 2, 4 and 6 h, and the levels of full‐length TSP1 and its cleavage products were assessed by immunoblotting. Specifically, full‐length TSP1 was detected in cell lysates, whereas TSP1‐derived fragments were detected in concentrated conditioned supernatants. As ADAMTS4 expression increased over time, the 68 kDa mature form also accumulated, reflecting ADAMTS4 maturation rather than serving as a direct measure of proteolytic activity. Concomitantly, full‐length TSP1 gradually decreased, whereas TSP1‐derived fragments in the supernatant accumulated in a time‐dependent manner (Figure 6H). Using an antibody recognizing the N‐terminal region of TSP1, we found that the most prominent cleavage product migrated at approximately 25 kDa. Previous studies have demonstrated that the 25 kDa N‐terminal fragment of TSP1, which possesses heparin‐binding capacity, retains substantial biological activity, including the ability to promote endothelial cell migration, angiogenesis and actin cytoskeletal disassembly.^21^ These observations suggest that the fragment detected in our system is likely to be biologically active, rather than merely an inert byproduct of protein degradation.

Because our earlier conditional knockout experiments had already demonstrated that *Adamts4* deficiency protects against fibrotic progression and cardiac dysfunction, we next turned to a gain‐of‐function model to more directly assess whether enhanced ADAMTS4‐TSP1 signalling aggravates pathological phenotypes. We therefore prioritized the use of Tcf21‐lineage fibroblast *Adamts4* overexpression (cTg), for which successful model generation had been validated previously (Figure 5B). Under the same LPS stimulation conditions, CFs isolated from cTg mice exhibited increased total ADAMTS4 expression, a reduced relative abundance of the 68 kDa mature band, and enhanced TSP1 fragmentation, as reflected by decreased full‐length TSP1 and accumulation of TSP1‐derived fragments (Figure 6I).

To further explore the functional significance of this phenomenon, we inhibited *Tsp1* expression in CFs using shRNA. Short hairpin RNA targeting *Tsp1* (*shTsp1*) effectively reduced TSP1 expression, as confirmed by Western blotting (Figure S7E). Functionally, *shTsp1* significantly improved the viability of CFs isolated from cTg mice following LPS stimulation, as assessed by CCK‐8 assay (Figure S7F). We then performed rescue experiments to determine whether TSP1 is required for ADAMTS4‐driven fibroblast activation. Six experimental groups were established, and fibrosis‐associated gene expression was evaluated by qPCR. TSP1 deficiency significantly attenuated ADAMTS4‐induced fibroblast activation, as evidenced by the marked suppression of *α‐SMA* and *Col1a1* mRNA expression in oeAdamts4‐treated cells following TSP1 knockdown (Figure 6J,K).

The above findings suggested that ADAMTS4 may function, at least in part, by cleaving TSP1. To further enhance the potential therapeutic relevance of our study, we evaluated the effect of an ADAMTS4‐neutralizing antibody during Adamts4 overexpression in HCFs. Notably, administration of the neutralizing antibody significantly attenuated the accumulation of TSP1 cleavage fragments in a concentration‐dependent manner (Figure S8A), supporting the notion that ADAMTS4 enzymatic activity is required for TSP1 processing in this context. To further assess whether TSP1 processing could also be detected at the cardiac tissue level in vivo, we performed Western blot analysis using whole‐heart lysates from Adamts4fl/fl mice under basal and LPS‐stimulated conditions. Consistent with the cleavage pattern observed in fibroblast‐based systems, LPS stimulation reduced full‐length TSP1 and increased an approximately 25 kDa TSP1‐immunoreactive fragment in whole‐heart lysates (Figure S8B). Because whole‐heart lysates contain multiple cardiac cell types and extracellular matrix components, this result provides tissue‐level evidence of TSP1 processing in LPS‐stimulated hearts, but does not by itself establish fibroblast‐specific or ADAMTS4‐specific TSP1 cleavage. In the above validations, immunoblot analysis consistently revealed a predominant TSP1 cleavage product at approximately 25 kDa. Based on this, we hypothesized that this fragment may represent a functionally relevant cleavage product contributing to pathological responses. Accordingly, we proceeded to investigate the role of TSP1 cleavage‐derived fragments in subsequent experiments.

### 2.4 ADAMTS4‐mediated cleavage of TSP1 at residues 236–246 drives fibroblast activation via TGF‐β/Smad and NF‐κB signalling

**FIGURE 7:**
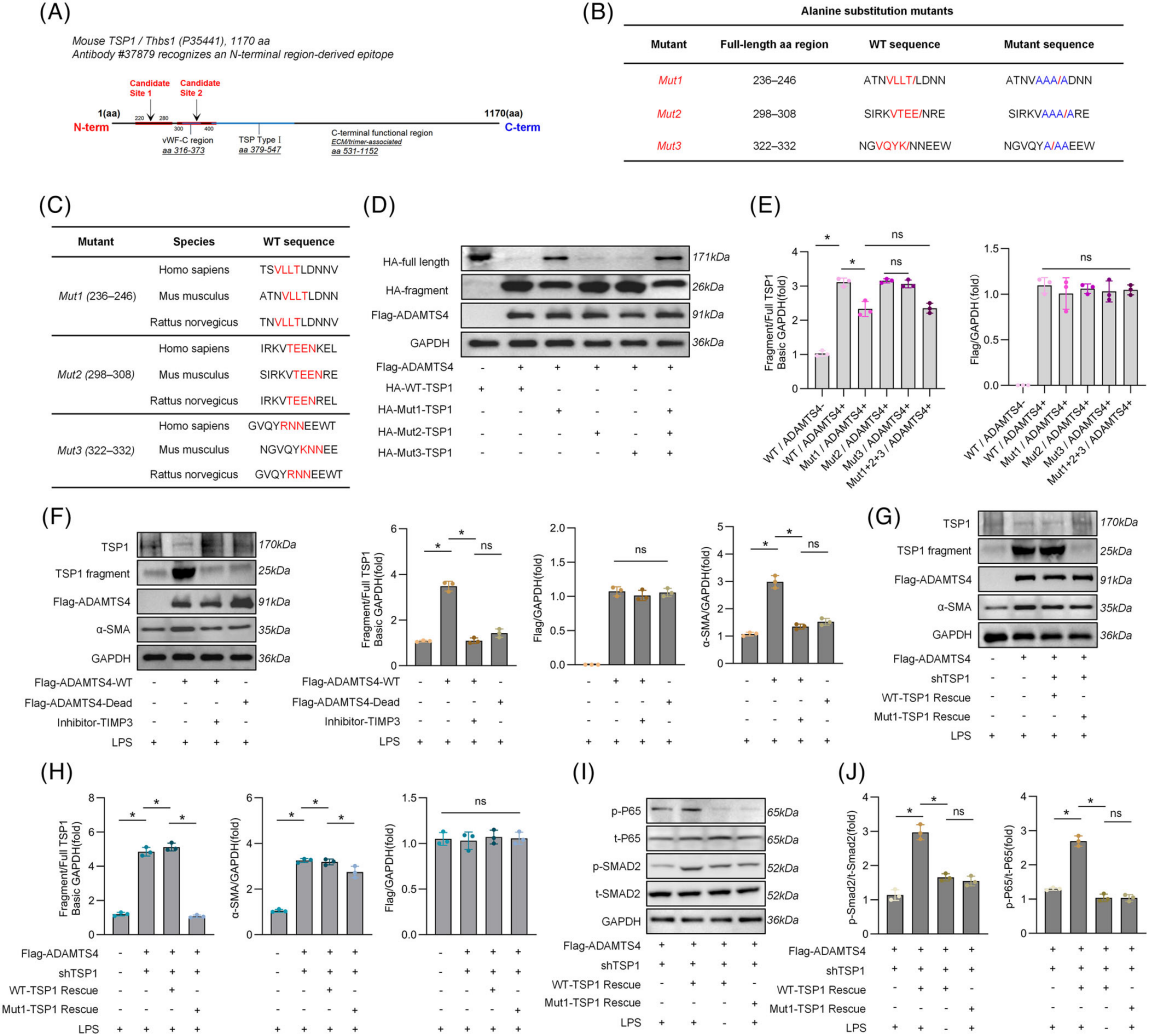
ADAMTS4‐mediated cleavage of TSP1 at residues 236–246 drives fibroblast activation via TGF‐β/Smad and NF‐κB signalling. (A) Schematic representation of full‐length mouse TSP1 highlighting predicted ADAMTS4 cleavage sites within the N‐terminal region. Two candidate cleavage regions (Site1 and Site2) identified by protease prediction analysis are indicated. (B) Alanine substitution strategy for candidate cleavage motifs within TSP1. Three regions selected based on prediction and structural accessibility (236–246 aa, 298–308 aa, and 322–332 aa) were mutated to alanine. (C) Sequence alignment of candidate cleavage regions across *Homo sapiens*, *Mus musculus* and *Rattus norvegicus*, demonstrating conservation of key residues. (D) Cleavage of TSP1 was assessed by Western blotting using antibodies against HA (full‐length and fragment), Flag (ADAMTS4), and GAPDH. (E) Full‐length intracellular proteins were normalized to GAPDH, whereas secreted TSP1‐derived fragments in conditioned media were normalized to total membrane protein staining. The resulting ratios were quantified and statistically analysed. (F) NIH/3T3 fibroblasts were transfected with wild‐type ADAMTS4 or the catalytically inactive ADAMTS4‐E362A mutant and subsequently stimulated with 10 µg/mL LPS for 48 h, with or without TIMP3 treatment. (G) NIH/3T3 fibroblasts subjected to shRNA‐mediated Tsp1 knockdown were reconstituted with HA‐tagged wild‐type TSP1 (WT‐TSP1) or the cleavage‐resistant Mut1‐TSP1 variant, co‐transfected with Flag‐tagged ADAMTS4, and subsequently stimulated with 10 µg/mL LPS for 48 h. Full‐length TSP1, ADAMTS4, α‐SMA and GAPDH in cell lysates, together with secreted TSP1‐derived fragments in concentrated conditioned media, were assessed by Western blotting. (H) Densitometric quantification of full‐length TSP1, secreted TSP1‐derived fragments, ADAMTS4, and α‐SMA from (G). Intracellular proteins were normalized to GAPDH, whereas secreted TSP1‐derived fragments were normalized to the total protein signal in the corresponding conditioned‐medium lane. (I) Western blot analysis of downstream signalling pathways in fibroblasts from (G), including phosphorylated and total Smad2 and NF‐κB p65. (J) Quantification of p‐Smad2/Smad2 and p‐p65/p65 ratios from (I). Data are presented as mean ± SEM. Normality and homogeneity of variance were assessed using Shapiro–Wilk and Levene's tests, respectively. Two‐group comparisons were performed using unpaired two‐tailed Student's *t*‐test or Mann–Whitney *U* test as appropriate. Multiple‐group comparisons were conducted using one‐way ANOVA with Tukey's post hoc test or Tamhane's T2 test; non‐parametric data were analysed using Kruskal–Wallis test with Dunn's post hoc correction. All tests were two‐sided, with *p* < .05 considered statistically significant. * *p* < .05; ns, not significant.

To delineate the structural determinants governing ADAMTS4‐mediated proteolysis of thrombospondin‐1 (TSP1), we first focused our analysis on the N‐terminal 400 amino acids of TSP1. This region was prioritized based on the reproducible enrichment of an approximately 25 kDa cleavage fragment detected by immunoblotting in ADAMTS4‐overexpressing cardiac fibroblast‐related systems (Figure 6H,I), which guided the candidate cleavage‐region mapping illustrated in Figure 7A. We employed the protease substrate prediction platform Prosperous Plus (http://prosperousplus.unimelb‐biotools.cloud.edu.au/index.php) to systematically scan the full‐length TSP1 sequence. Within the defined N‐terminal window, this analysis identified five high‐confidence candidate motifs with prediction scores ≥ 0.849. These predicted sites were further refined into five candidate regions, designated Mut1–5, based on structural accessibility (Figure S9A,B). To refine these candidates, we assessed their surface accessibility using AlphaFold‐derived three‐dimensional structural models. Candidate Mut1 (298–308 aa), Candidate Mut2 (236–246 aa), and Candidate Mut5 (322–332 aa) exhibited favourable solvent‐exposed topologies. In contrast, Candidate Mut3 (157–167 aa) was excluded due to its burial within a dense local conformation, and Candidate Mut4 (209–219 aa) was omitted owing to steric hindrance within a crowded surface microenvironment (Figure S9C). Integrating these structural insights, we selected the three accessible regions for functional interrogation and renamed them sequentially as Mut1 (236–246 aa), Mut2 (298–308 aa) and Mut3 (322–332 aa) (Figure 7B). Multiple sequence alignment revealed that Mut1 and Mut2 are highly conserved across mouse, rat, and human orthologues, while the Mut3 region retains a core recognition motif (Figure 7C).

To assess functional relevance, we employed a co‐expression system in NIH/3T3 fibroblasts expressing Flag‐tagged ADAMTS4 and HA‐tagged TSP1 (wild‐type or respective mutants). Immunoblot analysis demonstrated that the Mut1 single mutation was sufficient to markedly attenuate the generation of the TSP1 cleavage fragment. In contrast, individual mutations at Mut2 or Mut3 exhibited no discernible effect on proteolysis, and the triple mutant (Mut1+2+3) did not confer additive inhibition beyond that achieved with Mut1 alone (Figure 7D,E). These data establish the 236–246 aa segment (Mut1) as the requisite structural basis for ADAMTS4‐catalyzed TSP1 cleavage. We further corroborated the catalytic dependence of this event utilizing the inactive ADAMTS4‐E362A mutant and the broad‐spectrum ADAMTS inhibitor, tissue inhibitor of metalloproteinases 3 (TIMP3). In the context of lipopolysaccharide (LPS) stimulation, overexpression of wild‐type ADAMTS4 resulted in pronounced consumption of full‐length TSP1 concomitant with accumulation of the ∼25 kDa fragment and upregulation of α‐smooth muscle actin (α‐SMA). Crucially, this cleavage event and the corresponding increase in α‐SMA were abrogated by either the E362A mutation or TIMP3 treatment (Figure 7F). Collectively, these findings confirm that ADAMTS4‐mediated cleavage of TSP1 is strictly contingent upon its catalytic competence.

To investigate the downstream pathophysiological consequences of Mut1 cleavage, we performed rescue experiments in the context of shRNA‐mediated TSP1 knockdown. The silencing efficiency of shTsp1 in mouse cells had been validated previously. Cells were reconstituted with either wild‐type TSP1 (WT‐TSP1) or the cleavage‐resistant Mut1‐TSP1 variant and subsequently challenged with 10 µg/mL LPS for 48 h (Figure 7G,H). Reconstitution with WT‐TSP1 restored the appearance of the cleavage fragment and maintained elevated α‐SMA expression. Conversely, reconstitution with Mut1‐TSP1 resulted in diminished fragment generation and a corresponding reduction in α‐SMA levels. These observations imply that disrupting the Mut1 cleavage site mitigates the profibrotic phenotype driven by ADAMTS4‐mediated TSP1 proteolysis.

We next examined alterations in downstream signalling pathways associated with fibroblast activation. Prior evidence indicates that TSP1 facilitates the activation of latent transforming growth factor‐β (TGF‐β), thereby potentiating the canonical TGF‐β/Smad signalling axis.^31^ To determine whether TSP1 cleavage modulates TGF‐β activation, we analysed the conditioned media and lysates from the aforementioned co‐transfection systems under LPS‐stimulated and unstimulated conditions (Figure 7I,J; Figure S11). Quantification of TGF‐β1 in the supernatant revealed that, in the presence of LPS and Flag‐ADAMTS4, reconstitution with WT‐TSP1 significantly elevated the concentration of active TGF‐β1 and the ratio of active to total TGF‐β1. This effect was significantly blunted in the absence of LPS or upon reconstitution with the cleavage‐resistant Mut1‐TSP1 variant (Figure S11). Concordantly, immunoblot analysis of cell lysates demonstrated that reconstitution with Mut1‐TSP1 led to a marked decrease in the phosphorylation levels of Smad2 and the NF‐κB subunit p65 compared to WT‐TSP1 (Figure 7I,J).

Taken together, these results indicate that ADAMTS4‐mediated cleavage of TSP1 at the Mut1 region facilitates the activation of TGF‐β1 and pro‐inflammatory NF‐κB signalling within the inflammatory milieu induced by LPS. This mechanism contributes critically to the regulation of fibroblast activation. Therefore, the Mut1 domain not only constitutes the essential structural determinant for ADAMTS4‐dependent TSP1 proteolysis but also drives a profibrotic phenotype through the coordinated engagement of the TGF‐β/Smad and NF‐κB pathways.

## 3 DISCUSSION

Currently, therapeutic strategies for septic cardiomyopathy (SCM) primarily focus on acute cardiac injury, whereas effective clinical interventions specifically targeting sepsis‐induced myocardial fibrosis remain lacking.^32^ Defining the mechanisms that connect septic inflammation with maladaptive matrix remodelling may therefore reveal new therapeutic opportunities. In this study, we identify fibroblast‐lineage Adamts4 as a critical driver of LPS‐induced cardiac dysfunction and fibrotic remodelling. Mechanistically, our findings support a model in which ADAMTS4 cleaves thrombospondin‐1 (TSP1) within the 236–246 aa region, thereby promoting myofibroblast activation, TGF‐β/Smad and NF‐κB signalling, and pathological extracellular matrix (ECM) deposition. Although septic cardiac injury has traditionally been interpreted mainly through cardiomyocyte‐centred mechanisms, cardiac fibroblasts are increasingly recognized as active regulators of myocardial remodeling.^33^, ^34^ By sensing inflammatory and mechanical cues and communicating with cardiomyocytes and endothelial cells through paracrine signalling, fibroblasts shape both ECM remodelling and intercellular stress responses.^35^ Thus, fibroblast‐intrinsic regulators such as ADAMTS4 may provide therapeutic entry points for inflammation‐associated cardiac fibrosis. From a translational perspective, such fibroblast‐centred targets may complement current supportive strategies for SCM by addressing maladaptive matrix remodelling rather than acute hemodynamic dysfunction alone. Our findings should therefore be interpreted in the context of inflammation‐driven matrix dysregulation during septic injury progression, rather than chronic scar formation per se.

The ADAMTS family comprises secreted metalloproteinases with established roles in ECM remodelling. Recent studies have revealed distinct functions of ADAMTS members in cardiovascular pathology: ADAMTS7 deficiency ameliorates atherosclerosis and intimal remodelling through endothelial cell‐mediated effects,^36^ whereas ADAMTS5 promotes ECM remodelling and contributes to heart failure.^37^ Although ADAMTS proteases are broadly involved in ECM turnover,^38^, ^39^, ^40^ how individual family members act within specific cardiac lineages and disease settings remains less clear. Notably, αMHC‐Cre‐mediated deletion of ADAMTS8 or ADAMTS2 has been reported to exert opposing effects on cardiac hypertrophy,^41^, ^42^ further underscoring the context‐dependent functions of individual ADAMTS family members in cardiovascular disease. Our findings extend this concept to septic cardiac remodelling by identifying ADAMTS4 as a fibroblast‐enriched matrix protease that links inflammatory stress to pathological ECM remodelling. This lineage‐ and context‐dependent profile may be clinically relevant because selective modulation of disease‐activated matrix proteases could offer greater specificity than broad suppression of ECM turnover.

This lineage‐centred interpretation is further supported by using three complementary fibroblast‐lineage Cre drivers that capture distinct fibroblast states during cardiac remodelling. The tamoxifen‐inducible Tcf21‐iCreERT2 driver was used to manipulate Adamts4 in the Tcf21‐lineage fibroblast compartment, which includes resident cardiac fibroblasts relevant to the early stages of cardiac remodeling^43^; Col1a2‐CreER labels collagen‐producing fibroblasts across a broader fibrogenic stromal compartment^44^; and Postn‐CreER preferentially marks injury‐activated myofibroblasts involved in pathological matrix deposition.^45^ The consistent protective effects observed across these models suggest that ADAMTS4 contributes to fibroblast‐mediated remodelling across multiple activation states, rather than acting only within a single fibroblast subset. Complementing these loss‐of‐function models, Tcf21‐lineage fibroblast Adamts4 overexpression did not induce an overt basal fibrotic phenotype but markedly aggravated LPS‐induced cardiac dysfunction and fibrosis, indicating that the pathogenic activity of ADAMTS4 is stress‐dependent and requires an inflammatory microenvironment. In this regard, ADAMTS4 may function as a stimulus‐responsive amplifier of fibroblast activation, a concept consistent with previous observations that ADAMTS16 exacerbates cardiac fibrosis under pathological stress through TGF‐β‐related mechanisms.^46^ However, because ADAMTS4 is a secreted ECM‐associated protease and LPS induces systemic inflammation, contributions from circulating or extracardiac ADAMTS4 cannot be excluded. ELISA showed LPS‐induced increases in serum and cardiac ADAMTS4, with a greater relative increase in the heart. These data do not establish the source of circulating ADAMTS4. Nevertheless, together with isolated cardiac fibroblast analyses, primary‐cell experiments, and fibroblast‐lineage models, the findings support a cardiac fibroblast‐associated ADAMTS4‐TSP1 axis in LPS‐induced cardiac injury and fibrotic remodelling.

Our identification of TSP1 through immunoprecipitation‐mass spectrometry screening provides a mechanistic link between fibroblast‐derived matrix protease activity and downstream profibrotic signalling. These findings further suggest that ADAMTS4 promotes structurally selective TSP1 proteolytic processing with functional consequences. The recurrent detection of an approximately 25 kDa N‐terminal TSP1‐immunoreactive band guided our focus towards the N‐terminal region of TSP1. Because TSP1 is a heavily glycosylated matricellular glycoprotein, extensive post‐translational modifications may influence its electrophoretic mobility; therefore, this band should be interpreted as an indicative processing product rather than a strictly defined terminal cleavage fragment. By integrating Prosperous Plus‐based substrate prediction with AlphaFold‐based structural accessibility analysis, we prioritized the 236–246 aa region based on the convergence of prediction score, cross‐species conservation, solvent‐exposed topology, and functional validation by mutagenesis. These findings suggest that ADAMTS4 recognition of TSP1 depends not only on primary sequence motifs but also on local structural accessibility. Functionally, the comparison between cleavable wild‐type TSP1 and cleavage‐resistant Mut1‐TSP1 supports the concept that ADAMTS4‐mediated TSP1 processing is not merely degradative, but may generate a signal‐amplifying intermediate that promotes fibroblast activation, active TGF‐β1 release, and downstream Smad2 and p65 phosphorylation. Clinically, this raises the possibility that disease‐associated TSP1 fragments, rather than total TSP1 abundance alone, may better reflect pathogenic matrix remodelling activity in septic cardiac injury.

Given this functional interpretation of TSP1 processing, it is important to distinguish ADAMTS4 maturation from its actual proteolytic activity towards TSP1. Although the 68 kDa mature form of ADAMTS4 was quantified as an indicator of enzyme maturation, the mature/full‐length ADAMTS4 ratio should not be interpreted as a direct surrogate for TSP1‐directed proteolytic activity. ADAMTS4 activity in the extracellular space is likely regulated at multiple levels, including inhibition by endogenous regulators such as TIMP3, spatial restriction within the ECM, substrate accessibility, and post‐translational processing. These considerations may explain why mature ADAMTS4 abundance and TSP1 fragment generation do not necessarily follow a simple linear relationship. This context dependence is a recognized feature of ADAMTS‐mediated substrate processing. Beyond the catalytic domain, ancillary domains and exosite‐mediated interactions can critically determine substrate recognition, binding stability, and cleavage efficiency.^47^, ^48^ In addition, extracellular localization and substrate conformation may strongly influence whether a potential substrate is accessible to proteolysis. The ADAMTS13‐von Willebrand factor paradigm illustrates this principle, as efficient cleavage depends on conformational exposure of cryptic sites within a physiological microenvironment shaped by shear stress and cofactors.^49^ Therefore, although earlier cell‐free assays using non‐full‐length ADAMTS4 protein suggested that ADAMTS4 does not share the TSP1/2‐cleaving activity reported for ADAMTS1,^22^ such simplified biochemical systems may not fully capture proteolysis within an inflamed ECM‐rich cellular environment. In this study, the interaction, inhibition, time‐course, and mutagenesis data support ADAMTS4‐dependent TSP1 processing under LPS‐stimulated conditions, while also indicating that this event is governed by the local extracellular context rather than by ADAMTS4 maturation alone.

This extracellular regulation also places the ADAMTS4/TSP1 axis within the broader inflammatory and matrix‐remodelling microenvironment of sepsis. Systemic and myocardial inflammatory cues, including TNF‐α, IL‐1β, IL‐6, TGF‐β, oxidative stress and Toll‐like receptor signalling, may converge to induce Adamts4 expression in cardiac fibroblasts and thereby link acute inflammatory stimulation to sustained fibroblast activation and ECM remodelling. The inflammatory mediators increased in our model, including MCP‐1, IL‐6, TNF‐α and IL‐1β, are consistent with a fibroblast–immune communication network in which inflammatory cells and activated fibroblasts may mutually reinforce local cytokine production and matrix remodelling. Within this framework, NF‐κB should be viewed as a representative downstream indicator connecting ADAMTS4/TSP1‐dependent ECM remodelling with inflammatory amplification, rather than as a comprehensive assessment of all inflammatory signalling pathways. The ECM itself may also shape this process, as sepsis‐associated changes in matrix composition and stiffness, particularly involving fibronectin, periostin, and collagen, could influence TSP1 accessibility, proteolytic efficiency, and downstream integrin/TGF‐β signalling. Future studies combining cytokine perturbation, immune‐cell profiling, and matrix‐focused analyses will be needed to define which upstream inflammatory and stromal cues are most responsible for Adamts4 induction and TSP1 cleavage in vivo.

In summary, this study establishes *Adamts4* as a central regulatory node in sepsis‐induced cardiac inflammation, oxidative injury, and myocardial fibrosis through its site‐specific proteolytic cleavage of TSP1. The 236–246 aa TSP1 region provides a structure–function basis for understanding how cleavage‐dependent matrix signalling promotes fibroblast activation and downstream TGF‐β/Smad and NF‐κB signalling. Targeting ADAMTS4 activity, limiting pathogenic TSP1 processing, or blocking cleavage‐dependent downstream signalling may therefore represent complementary therapeutic strategies for septic cardiomyopathy and other inflammation‐driven fibrotic diseases.

## 4 METHODOLOGY

### 4.1 Study Design

All animal procedures complied with the NIH Guide for the Care and Use of Laboratory Animals (2011 revision) and were approved by the Animal Care and Use Committee of Renmin Hospital of Wuhan University. The study was designed to define the role of *Adamts4* in pathological cardiac fibrosis. Animal numbers were selected based on previous experimental experience. Investigators were blinded to group allocation, and no samples were excluded from the analyses. Sample size, biological replicates and independent experimental numbers are provided in the corresponding figure legends.

### 4.2 Animals and Treatments

We used genetically engineered mouse models to investigate the cell type‐specific functions of ADAMTS4 in cardiac fibrosis. The conditional knockout allele Adamts4^fl/fl^ (C57BL/6JGpt‐Adamts4^em1Cflox^/Gpt, Strain #T019456) was obtained from GemPharmatech Co., Ltd.

Tamoxifen‐inducible Tcf21‐iCreERT2 mice (C57BL/6‐*Tcf21^tm1(icre/ERT2)Bcgen/Bcgen^*) were purchased from Biocytogen. Tamoxifen‐inducible Col1a2‐CreER mice (B6.Cg‐Tg^(Col1a2‐cre/ERT,‐ALPP)7Cpd/2J^) and Postn‐CreER mice (*Postn* ^tm2.1(cre/Esr1)Jmol^) were acquired from The Jackson Laboratory (Bar Harbor, ME, USA). Tcf21‐lineage Adamts4‐deficient mice, referred to as Adamts4 cKO mice, were generated by crossing Adamts4^fl/fl^ mice with Tcf21‐iCreERT2 mice. Col1a2‐lineage Adamts4‐deficient mice, referred to as Adamts4 cfKO mice, were generated by crossing Adamts4^fl/fl^ mice with Col1a2‐CreER mice. To induce recombination in the tamoxifen‐inducible Tcf21‐iCreERT2 and Col1a2‐CreER models, adult mice received intraperitoneal injections of tamoxifen (25 mg/kg/day, Sigma‐Aldrich) for five days. A 2‐week tamoxifen washout period was allowed before LPS administration or tissue collection. Knockout efficiency was confirmed by assessing ADAMTS4 protein levels in isolated cardiac fibroblasts (CFs) via Western blotting. For the Postn‐CreER‐mediated Adamts4 mfKO model, to enhance the specificity of Cre recombinase expression in activated myofibroblasts, mice received the same 5‐day intraperitoneal tamoxifen course and were also maintained on tamoxifen citrate‐containing diet (400 mg/kg, Envigo) from the day of injury induction until sacrifice to improve recombination in activated myofibroblasts. These procedures ensured reliable fibroblast‐lineage manipulation of Adamts4 expression in the corresponding Cre‐driver models.

Cardiomyocyte‐specific ADAMTS4 knockout mice (Adamts4 cmKO) were generated by crossing Adamts4^fl/fl^ mice with αMHC‐MerCreMer transgenic mice (B6.FVB(129)‐A1cf^Tg(Myh6‐cre/Esr1*)1Jmk/J^, The Jackson Laboratory). Endothelial cell‐specific ADAMTS4 knockout mice (Adamts4 ecKO) were produced by crossing Adamts4^fl/fl^ mice with Tie2‐iCreERT2 mice (B6.FVB‐Tg^(Tek‐icre/ERT2)1Soff/J^, The Jackson Laboratory). To induce Cre‐mediated recombination, adult Adamts4 ecKO mice and their corresponding littermate controls received intraperitoneal tamoxifen at 25 mg/kg/day for five consecutive days. A 2‐week tamoxifen washout period was allowed before subsequent experiments. Cre activation protocols for these strains followed the standard recommendations provided by The Jackson Laboratory. These models allowed assessment of ADAMTS4 function in non‐fibroblast cardiac cell populations.

To further delineate the role of ADAMTS4 in cardiac fibrosis, we employed a Tcf21‐lineage fibroblast Adamts4 conditional overexpression model (Adamts4 cTg). The *Adamts4* conditional overexpression mice (Adamts4 Tg^fl/fl^) were custom‐generated by GemPharmatech Co., Ltd. To generate the Tcf21‐lineage Adamts4 conditional overexpression model, Adamts4 Tg^fl/fl^ mice were crossed with B‐Tcf21‐iCreERT2 mice. Adult Adamts4 cTg mice and their corresponding littermate controls received intraperitoneal tamoxifen at 25 mg/kg/day for five consecutive days to induce iCreERT2‐mediated recombination and conditional Adamts4 overexpression in Tcf21‐lineage cells. A 2‐week tamoxifen washout period was allowed before subsequent experiments. Adamts4 overexpression was confirmed by Western blot analysis of cardiac fibroblasts isolated from Adamts4 cTg mice. Some of these mouse models have been previously characterized and used in studies by our collaborators.^50^

To preclude potential confounding effects of oestrogen, all experiments were conducted using male mice. For the LPS‐induced injury model, mice received intraperitoneal injection of LPS at 5 mg/kg once weekly for two consecutive weeks. Control mice were administered the same volume of sterile saline intraperitoneally on the same schedule. Different LPS regimens were applied according to the experimental objective. For preliminary short‐term phenotypic validation, mice were challenged with 10 mg/kg LPS for 24 h to assess cardiac ADAMTS4 expression. Subsequent functional studies were performed using two refined LPS protocols: an acute high‐dose challenge with 15 mg/kg LPS for survival analysis, and a repeated low‐dose regimen consisting of 5 mg/kg LPS administered intraperitoneally once weekly for two consecutive weeks to evaluate cardiac dysfunction, myocardial injury, and fibrotic remodelling. These LPS regimens were designed for distinct experimental purposes and should not be interpreted as directly interchangeable models. Nevertheless, the consistent direction of Adamts4 induction and ADAMTS4‐associated pathological changes across these complementary settings supports the robustness of the ADAMTS4 response to LPS‐induced inflammatory stress. All mice were housed under specific pathogen‐free (SPF) conditions at a controlled temperature of 25°C and 50% humidity, with a standardized 12‐h light/dark cycle. Mice had ad libitum access to food and water and were housed at a density of five animals per cage.

### 4.3 Echocardiography

Transthoracic echocardiography was performed in Adamts4 cKO, cfKO, mfKO, cmKO, ecKO and cTg mice, as well as their corresponding littermate controls. Transthoracic echocardiography was performed on Days 7 and 14 after the first LPS administration. Mice were anaesthetized by inhalation of 2% isoflurane and placed in a supine position for cardiac imaging. Cardiac morphology and systolic function were evaluated using a Vevo® 3100 high‐resolution preclinical ultrasound system (FUJIFILM VisualSonics) equipped with a 30‐MHz linear‐array transducer. The measured echocardiographic indices included ejection fraction (EF), fractional shortening (FS), left ventricular end‐systolic volume (LVESV), left ventricular end‐diastolic volume (LVEDV), left ventricular end‐systolic diameter (LVESD), interventricular septal thickness during systole and diastole (IVSs and IVSd), and left ventricular posterior wall thickness during systole and diastole (LVPWs and LVPWd). For each mouse, values were calculated from 3 to 4 consecutive cardiac cycles to improve measurement consistency. During image acquisition, compression of the thoracic wall was carefully minimized to avoid artificial alterations in ventricular chamber geometry and to ensure accurate assessment of cardiac functional parameters.

### 4.4 Histological Analysis and Immunofluorescence Staining

Hearts were arrested in 10% KCl, fixed in 10% neutral formalin, dehydrated, embedded in paraffin, and sectioned. Haematoxylin and eosin (H&E) staining was performed to evaluate myocardial fibre morphology; Picrosirius red (PSR) staining was used to quantify the collagen‐positive fibrotic area, and values were averaged across multiple randomly selected, non‐overlapping fields. To determine the cardiac localization of *Adamts4*, tissue sections were incubated overnight at 4°C with primary antibodies against ADAMTS4 (1:200, #ab185722, Abcam) and α‐SMA (1:200, #ab8295, Abcam). After washing, sections were exposed for 1 h at room temperature to species‐matched fluorescent secondary antibodies, including Alexa Fluor 568‐conjugated goat anti‐rabbit IgG (H+L; Invitrogen) and Alexa Fluor 488‐conjugated goat anti‐mouse IgG (H+L). The reliability of ADAMTS4 immunostaining was confirmed using tissues or CFs with conditional *Adamts4* deficiency. Myocardial superoxide generation was evaluated by staining fresh heart sections with dihydroethidium (DHE, 1 µM) for 30 min. Cell apoptosis in myocardial sections was assessed using a terminal deoxynucleotidyl transferase‐mediated dUTP nick‐end labelling (TUNEL) kit (Millipore, USA). For selected samples, wheat germ agglutinin (WGA, 1:100) staining was performed at 37°C for 2 h, and nuclei were counterstained with DAPI‐containing SlowFade™ Gold antifade reagent (#S36939, Invitrogen). Cardiomyocyte cross‐sectional area was calculated from more than 30 cells per sample. Images were captured with an Olympus DP72 digital microscope system and quantified using ImageJ software.

### 4.5 RNA‐seq Analysis

Hearts were harvested 12 h after intraperitoneal administration of 15 mg/kg LPS. Total RNA was isolated from samples and assessed for integrity using a Bioanalyzer 2100 system (Agilent Technologies). Separate libraries were prepared for non‐strand‐specific RNA‐seq. For non‐strand‐specific libraries, mRNA was enriched using poly‐T magnetic beads, fragmented, and reverse‐transcribed into first‐strand cDNA with random hexamers, followed by second‐strand synthesis, end repair, A‐tailing, adapter ligation, size selection, amplification, and purification. All libraries were quantified using Qubit and real‐time PCR, and fragment size distributions were assessed on a Bioanalyzer to ensure quality control.

Qualified RNA‐seq libraries were pooled according to concentration and target data yield and sequenced on an Illumina platform using a “sequencing‐by‐synthesis” approach. Fluorescent signals were captured and converted to sequence reads. Raw fastq files were processed with fastp to remove adapters, poly‐N, and low‐quality reads, producing clean datasets. Q20, Q30 and GC content metrics were calculated. Reference genome indices were generated with Hisat2 v2.0.5, and paired‐end reads were aligned to the genome. Gene‐level read counts were obtained using featureCounts v1.5.0‐p3, and FPKM values were computed to quantify expression levels. These data were then used for downstream expression and pathway analyses.

Differential expression was analysed using DESeq2 for datasets with biological replicates. Genes with adjusted *p* ≤ .05 and |log2(fold change)| ≥ 1 were considered significantly differentially expressed. GO and KEGG enrichment analyses were performed with the clusterProfiler R package, and GSEA was conducted using the GSEA tool. Protein‐protein interaction (PPI) analysis was performed based on the STRING database. RNA‐seq and related analyses were conducted by Novogene. This workflow provided a comprehensive framework for identifying sepsis‐related transcriptional changes in cardiac tissues.

### 4.6 Publicly Available Transcriptomic Dataset Analysis

Publicly available bulk and single‐cell transcriptomic datasets were obtained from the National Center for Biotechnology Information Gene Expression Omnibus (NCBI GEO). Four independent bulk transcriptomic datasets, GSE79962, GSE142615, GSE229925 and GSE266124, were used to evaluate the reproducibility of Adamts4/ADAMTS4 expression changes across human, mouse, and rat models of septic cardiomyopathy.

GSE79962 is a human myocardial expression microarray dataset generated using the Affymetrix Human Gene 1.0 ST Array platform (GPL6244). The present analysis included myocardial samples from 20 patients with sepsis and 11 non‐failing donor hearts. Samples from patients with ischemic heart disease or non‐ischemic dilated cardiomyopathy were not included in the present comparison. GSE142615 is a mouse cardiac microarray dataset generated using platform GPL27951 and contains heart samples collected 6 h after intraperitoneal administration of saline or 10 mg/kg LPS (*n* = 4 per group). GSE229925 is a mouse left ventricular bulk RNA‐seq dataset generated using the Illumina NovaSeq 6000 platform (GPL24247). Samples were collected 24 h after caecal ligation and puncture and included sham mice (*n* = 4), mice with high LVEF (LVEF ≥90%; *n* = 4), low LVEF (LVEF < 65%; *n* = 3), and normal LVEF (65% ≤ LVEF < 90%; *n* = 4). GSE266124 is a rat myocardial RNA‐seq dataset generated using the DNBSEQ‐T7 platform (GPL31008) and includes control, LPS and LPS plus calcitonin gene‐related peptide groups (*n* = 5 per group). Only the control and LPS groups were included in the present analysis

Cell‐cycle scores were calculated using established S‐phase and G2/M‐phase gene sets. Gene‐expression counts were normalized and variance‐stabilized using SCTransform with the glmGamPoi method, while mitochondrial transcript percentage, S‐phase score and G2/M‐phase score were regressed out. Principal component analysis was subsequently performed, and the first 40 principal components were used to construct the shared nearest‐neighbour graph. Candidate clustering resolutions of 0.4, 0.6, 0.8, 1.0 and 1.4 were evaluated, and the clustering solution used for final cell‐type annotation contained 23 transcriptionally distinct clusters. Uniform Manifold Approximation and Projection was used for two‐dimensional visualization.

Cluster‐specific marker genes were identified using the FindAllMarkers function, with a minimum expression fraction of 0.25 and a log2 fold‐change threshold of 0.25. Cell populations were annotated according to cluster‐enriched genes and established cardiac lineage markers. Fibroblasts were identified by expression of Dcn, Col1a1, Col1a2 and Pdgfra; endothelial cells by Pecam1, Cdh5, Kdr; cardiomyocytes by Tnnt2, Myh6, Myh7 and Actc1; macrophages by Adgre1, C1qa, C1qb and Lyz2; and mural cells, including pericytes and vascular smooth muscle cells, by Rgs5, Pdgfrb, Cspg4, Acta2 and Tagln. Adamts4 expression was visualized across the annotated cell populations using feature plots and group‐specific UMAP plots. Because the comparison included one sham and one sepsis biological sample, differences in cell‐type abundance and the distribution of Adamts4‐positive cells were interpreted descriptively and were not subjected to biological replicate‐based inferential statistical testing.

### 4.7 Western Blotting and Quantitative Real‐Time PCR (qRT‐PCR)

Heart tissues and cultured cells were lysed in RIPA lysis buffer supplemented with protease and phosphatase inhibitors. Nuclear and cytoplasmic protein fractions were prepared using a Nuclear and Cytoplasmic Protein Extraction Kit (P0027, Beyotime Biotechnology) according to the manufacturer's instructions when nuclear pathway‐related proteins were examined. Conditioned media were collected, centrifuged to remove cell debris, and concentrated for the detection of secreted TSP1‐derived fragments. Protein concentrations were determined using a BCA protein assay kit. Equal amounts of protein were separated by SDS‐PAGE and transferred onto PVDF membranes. After blocking with 5% non‐fat milk in TBST at room temperature for 1 h, membranes were incubated with primary antibodies overnight at 4°C. The primary antibodies used in this study included antibodies against ADAMTS4, TSP1/THBS1, α‐SMA, GAPDH, Bcl‐2, Bax, Caspase‐3, cleaved Caspase‐3, IKKβ, phospho‐IKKβ, IκBα, phospho‐IκBα, NF‐κB p65, phospho‐NF‐κB p65, Smad2, phospho‐Smad2, HA‐tag and Flag/DDDDK‐tag.

After washing, membranes were incubated with the corresponding HRP‐conjugated secondary antibodies and visualized using an enhanced chemiluminescence detection system. Band intensities were quantified using ImageJ software. Phosphorylated proteins were normalized to their corresponding total proteins, intracellular proteins were normalized to GAPDH, and secreted TSP1‐derived fragments in conditioned media were normalized to the Ponceau S total‐protein signal in the corresponding membrane lane. Detailed antibody information, including suppliers, catalogue numbers and working dilutions, is provided in Table S1.

For densitometric quantification, protein band images were analysed using ImageJ software (National Institutes of Health). Briefly, images were converted to 8‐bit grayscale, regions of interest (ROIs) were delineated around each band, and background subtraction was performed using the rolling‐ball algorithm with a radius of 50 pixels. The integrated density of each band was measured. Relative intracellular protein levels were normalized to GAPDH or the corresponding total protein, whereas secreted TSP1‐derived fragments were normalized to membrane total protein staining as described above. Data are presented as fold changes relative to the control samples.

For RT‐qPCR, total RNA was isolated from tissues or cells, and cDNA was synthesized using the OneStep Ahead RT‐PCR Kit (Qiagen). Target mRNAs were quantified with LightCycler 480 SYBR Green Master Mix (Roche Diagnostics GmbH) on a real‐time PCR instrument. Each sample was measured in triplicate, and GAPDH served as the internal reference. Relative expression was calculated with the 2^−ΔΔCt^ method after normalization to GAPDH and comparison with the indicated control group. Primer sequences are provided in Table S2. No‐template controls and melting‐curve analyses were included in each run to verify amplification specificity and exclude potential contamination.

### 4.8 Cell culture, transfection and treatments

Different in vitro stimulation durations were used according to the experimental purpose: 24 h LPS stimulation was used for early ADAMTS4 expression validation and CCK‐8‐based viability assays, whereas 48 h LPS stimulation was used for fibroblast activation, TSP1 cleavage, TGF‐β1 activation, and rescue experiments. For TSP1 processing time‐course experiments, samples were collected at 0, 2, 4 and 6 h after LPS stimulation.

Primary adult mouse cardiac fibroblasts (CFs) were isolated following a previously established protocol. In brief, hearts were excised from 6‐ to 8‐week‐old mice under 2% isoflurane anaesthesia and carefully mounted on a Langendorff perfusion system. Initially, the hearts were perfused with pre‐warmed Hanks’ balanced salt solution to completely remove residual blood, and subsequently digested enzymatically in a solution containing hyaluronidase and collagenase II for 25 min, until the ventricular tissue became soft and loosely structured. The digested tissue was then finely minced and passed through a 200‐mesh cell strainer to obtain a single‐cell suspension. This suspension was incubated at 37°C for 1 h, allowing differential adhesion to selectively enrich CFs while minimizing contamination from other cardiac cell types. The purified fibroblasts were seeded into culture dishes and maintained in DMEM/F12 medium supplemented with 10% foetal bovine serum under humidified conditions at 37°C with 5% CO_2_, ensuring optimal growth and viability. All subsequent experiments were conducted using cells at Passage 1 or Passage 2 to preserve their phenotypic characteristics and functional integrity throughout the study. All procedures were performed under standardized conditions.

For LPS‐induced stimulation, purified CFs were serum‐starved for 12 h when required and then treated with 10 µg/mL LPS (#L2880, Sigma‐Aldrich) for 48 h to simulate the fibrotic phenotype. Before collection of conditioned media for TSP1 fragment detection, cells were maintained under serum‐free conditions during the stimulation period, and conditioned media were collected for subsequent concentration and immunoblot analysis.

To manipulate Adamts4 expression in primary mouse cardiac fibroblasts (CFs), loss‐ and gain‐of‐function approaches were performed separately. For Adamts4 knockdown, CFs were transfected with an shRNA construct targeting mouse Adamts4 (shAdamts4) or a corresponding non‐targeting control shRNA (shCtrl). After transfection and recovery, the cells were stimulated with 10 µg/mL LPS for the indicated duration. Knockdown efficiency was confirmed by Western blotting before subsequent functional analyses. For Adamts4 overexpression, CFs were transduced with a lentiviral vector encoding the full‐length coding sequence of mouse Adamts4 (oeAdamts4) or the corresponding control lentivirus (oeCtrl). Following lentiviral transduction, Adamts4 overexpression was verified by Western blotting, and the cells were subsequently treated with 10 µg/mL LPS for the indicated experiments.

To assess the role of TSP1 in ADAMTS4‐dependent fibrotic responses, CFs from Adamts4 Tg^fl/fl^ mice were infected with adenovirus carrying shRNA targeting mouse *Tsp1* (sh*Tsp1*). The adenoviral vectors, shRNAs, and corresponding control vectors were constructed and packaged by Wuhan GeneCreate Biological Engineering Co., Ltd.

For ADAMTS4 neutralization experiments, human primary cardiac fibroblasts (HCFs) were transduced with ADAMTS4‐overexpressing vector. During LPS stimulation, cells were treated with an ADAMTS4‐neutralizing monoclonal antibody, Human ADAMTS4 MAb clone 416610 (R&D Systems/Bio‐Techne, Cat. No. MAB4307; mouse IgG2A), at final concentrations of 20 or 40 µg/mL. This antibody is listed by the manufacturer for neutralization applications and has been validated to inhibit recombinant human ADAMTS4‐mediated cleavage of recombinant human aggrecan G1‐IGD‐G2 domain. Conditioned media were collected, centrifuged to remove cellular debris, and concentrated under identical experimental conditions. Equal amounts of conditioned‐medium protein, as determined using the BCA assay, were separated by SDS‐PAGE and transferred onto PVDF membranes. Following transfer, total protein in each membrane lane was visualized using Ponceau S staining. The integrated total‐protein signal of each lane was used as the loading control for conditioned‐medium samples. The band intensity of secreted TSP1‐derived fragments was normalized to the total protein signal in the corresponding conditioned‐medium lane. Secreted TSP1‐derived fragments were quantified independently from intracellular full‐length TSP1 and were not normalized to GAPDH or to intracellular full‐length TSP1.

NIH/3T3 mouse embryonic fibroblasts were provided by the institutional experimental platform and cultured in complete DMEM medium under standard incubator conditions at 37°C with 5% CO_2_. For plasmid transfection experiments, cells were seeded in culture plates and transfected when cell confluence reached approximately 75%. After plasmid transfection, cells were switched to serum‐free DMEM for LPS stimulation and conditioned medium collection when TSP1‐derived fragments were examined. Plasmid transfection was performed using Lipo3000 transfection reagent from Vazyme according to the manufacturer's protocol. For a 6‐well plate, a total of approximately 2.5 µg plasmid DNA was used per well, and the total amount of transfected DNA was kept constant among groups by adding the corresponding empty vector when necessary. After 6 h of transfection, the medium was replaced with fresh serum‐free or complete medium according to the experimental purpose. For experiments designed to detect secreted TSP1‐derived fragments, cells were maintained under serum‐free conditions during the stimulation period.

For mechanistic plasmid‐based experiments, NIH/3T3 cells were co‐transfected with Flag‐ADAMTS4 expression plasmid and HA‐tagged TSP1 plasmids, including WT‐TSP1 or the indicated mutant constructs. For TSP1 cleavage‐site analysis, equal molar amounts of HA‐tagged WT‐TSP1, Mut1‐TSP1, Mut2‐TSP1, Mut3‐TSP1, or Mut1+2+3‐TSP1 plasmids were used across groups. Under identical transfection conditions, the occurrence and extent of TSP1 cleavage were compared horizontally among different TSP1 constructs by detecting full‐length HA‐TSP1 in cell lysates and TSP1‐derived fragments in conditioned media. Because independent evaluation of the transfection efficiency of each mutant construct was technically limited, comparisons were performed under equal molar plasmid input, and HA‐tagged full‐length TSP1 together with Flag‐tagged ADAMTS4 expression was used as an internal indicator of successful transfection in the corresponding immunoblot analyses.

For pharmacological inhibition of ADAMTS4 activity, active recombinant mouse TIMP3 protein (Mus musculus; USCN; No. UAPA129Mu01) was used at a final concentration of 100 nM, corresponding to approximately 2.5 µg/mL. After plasmid transfection, cells were switched to serum‐free DMEM and treated with 10 µg/mL LPS in the presence or absence of TIMP3 for 48 h. TIMP3 was added simultaneously with LPS to inhibit ADAMTS4‐mediated proteolytic activity during inflammatory stimulation. Conditioned media and cell lysates were then collected for detection of TSP1‐derived fragments, full‐length TSP1, ADAMTS4, α‐SMA, and downstream signalling proteins.

For rescue experiments, TSP1‐depleted cells were reconstituted with HA‐tagged WT‐TSP1 or cleavage‐resistant Mut1‐TSP1 plasmids before LPS stimulation. Unless otherwise indicated, mechanistic NIH/3T3 experiments were stimulated with 10 µg/mL LPS for 48 h after plasmid transfection. When TSP1‐derived fragments were examined in conditioned media, cells were maintained under serum‐free conditions throughout the LPS stimulation period.

### 4.9 Cardiac Fibroblast Viability Assay

At 48 h after shRNA transfection or lentiviral transduction, CFs were switched to the indicated culture conditions and treated with PBS or 10 µg/mL LPS as required. Cell viability was assessed using the CCK‐8 assay at 0, 6, 12 and 24 h after treatment. At each indicated time point, 10 µL CCK‐8 reagent (#A311‐01, Vazyme) was added to each well and incubated at 37°C for 2 h. Absorbance at 450 nm was measured using a microplate reader. The experiment included three independent biological replicates, with six technical replicates per group.

### 4.10 Co‐Immunoprecipitation‐Mass Spectrometry (Co‐IP‐MS), Co‐Immunoprecipitation (Co‐IP) and GST Pull‐Down Assays

#### 4.10.1 Co‐IP‐MS

Heart lysates were incubated overnight at 4°C with Protein A/G beads pre‐conjugated with anti‐ADAMTS4 antibody or control IgG to collect immune complexes, which were then washed. Immunoprecipitated complexes were eluted, separated by SDS‐PAGE, and visualized by protein staining. Gel bands were excised, enzymatically digested, purified using a C18 column, and analysed using an Orbitrap Astral mass spectrometer. Parallel immunoprecipitated samples were analysed by Western blotting to verify ADAMTS4 enrichment and candidate interacting proteins. Data were acquired in data‐dependent acquisition (DDA) mode, which provides high‐quality tandem mass spectra and enables confident identification of low‐abundance interacting proteins, making it particularly suitable for CoIP‐MS‐based interaction screening. The acquired MS data were analysed using Proteome Discoverer software against the Uniprot mouse reference proteome.

#### 4.10.2 Co‐IP

LPS‐treated mouse heart tissues were washed twice with ice‐cold PBS and weighed. Lysis/wash buffer supplemented with protease inhibitors was added at a ratio of 10–20 µL/mg tissue. Tissues were homogenized and lysed on ice for 30 min, followed by centrifugation at 12 000 rpm, approximately 13 800 × g, at 4°C for 10 min. The supernatants were collected, and 500–1000 µg of total protein was incubated overnight at 4°C with 2–10 µg anti‐ADAMTS4 antibody, anti‐TSP1 antibody, or corresponding isotype IgG control in a final volume of 500 µL. Protein A/G magnetic beads were washed twice with lysis/wash buffer, added to the antibody‐lysate mixture, and incubated with rotation at 4°C for 1–2 h. After magnetic separation, the beads were washed three times with lysis/wash buffer. Bound protein complexes were eluted by adding 25–50 µL 1× SDS‐PAGE loading buffer and heating at 95°C for 5 min. Eluted proteins were analysed by SDS‐PAGE and Western blotting. ADAMTS4 immunoprecipitates were immunoblotted for TSP1, and reciprocal TSP1 immunoprecipitates were immunoblotted for ADAMTS4.

#### 4.10.3 GST Pull‐Down

For GST pull‐down assays, Rosetta (DE3) *E. coli* cells were transformed with the pGEX‐6p‐1‐GST‐ADAMTS4 expression vector or the corresponding GST control vector. Protein expression was induced with 0.5 mM IPTG, and bacterial lysates were incubated with glutathione‐agarose 4B beads (#17075601, GE Healthcare) at 4°C for 1 h to immobilize GST‐tagged proteins. After extensive washing, bead‐bound GST‐ADAMTS4 or GST control proteins were incubated with purified His‐tagged TSP1 at 4°C for 6 h. The beads were then washed to remove unbound proteins, and bound complexes were eluted with SDS‐PAGE loading buffer and analysed by Western blotting using anti‐His and anti‐GST antibodies.

### 4.11 Plasmid construction and mutagenesis

N‐terminal HA‐tagged full‐length mouse TSP1 mammalian expression plasmid and C‐terminal Flag‐tagged full‐length mouse ADAMTS4 mammalian expression plasmid were constructed by Shanghai GenePharma Co., Ltd. To prioritize candidate TSP1 mutant sequences, the full‐length mouse TSP1 sequence was first analysed using the Prosperous Plus protease substrate prediction platform. Because immunoblotting consistently detected an approximately 25 kDa N‐terminal TSP1‐immunoreactive fragment in ADAMTS4‐overexpressing fibroblast systems, the N‐terminal 400 amino acids of TSP1 were selected for detailed candidate mapping. Within this region, predicted motifs with scores ≥ 0.849 were retained and expanded into five 11‐amino‐acid candidate regions. These regions were then prioritized according to three criteria: prediction score, AlphaFold‐based solvent‐exposed structural accessibility, and cross‐species conservation among mouse, rat and human TSP1. Candidate regions that were buried within dense local structures or located in sterically constrained regions were excluded. Based on this prioritization workflow, three accessible regions were selected for site‐directed mutagenesis and renamed according to their order in the TSP1 sequence as Mut1 (236‐246 aa), Mut2 (298‐308 aa), and Mut3 (322‐332 aa). Single‐site TSP1 mutants, including HA‐Mut1‐TSP1, HA‐Mut2‐TSP1, and HA‐Mut3‐TSP1, were generated from the WT HA‐TSP1 plasmid using a site‐directed mutagenesis kit from Beyotime Biotechnology according to the manufacturer's protocol (#D0206M). The triple‐mutant construct HA‐Mut1+2+3‐TSP1, carrying simultaneous mutations in all three predicted regions, was directly generated by Shanghai GenePharma Co., Ltd.

For ADAMTS4 enzymatic activity analysis, the catalytically inactive mutant Flag‐ADAMTS4‐E362A was generated from the WT Flag‐ADAMTS4 plasmid by substituting glutamic acid at residue 362 with alanine using the Beyotime site‐directed mutagenesis kit. WT Flag‐ADAMTS4 and catalytically inactive Flag‐ADAMTS4‐E362A were used to distinguish catalytic activity‐dependent effects on TSP1 cleavage. All plasmid constructs and mutations were verified by Sanger sequencing provided by the institutional research platform of the hospital (Figure S10A–D). The sequences are available upon reasonable request.

### 4.12 Measurement of ADAMTS4 and active/total TGF‐β1 by ELISA

Serum and cardiac tissue ADAMTS4 levels were measured using a Mouse ADAMTS4 ELISA Kit (RK08749; ABclonal) according to the manufacturer's instructions. Blood samples were allowed to clot and centrifuged to obtain serum. Cardiac tissues were homogenized in lysis buffer containing protease inhibitors and centrifuged to remove insoluble material. Total protein concentrations in cardiac homogenates were determined by BCA assay. Serum and cardiac samples were diluted as required to fall within the standard‐curve range and analysed in duplicate. After incubation with detection antibody, streptavidin‐HRP, and TMB substrate, absorbance was measured at 450 nm. Serum ADAMTS4 concentrations were expressed as ng/mL, whereas cardiac ADAMTS4 content was normalized to total protein and expressed as pg/mg total protein.

For TGF‐β1 measurements, conditioned media were collected from NIH/3T3 cells transfected with Flag‐ADAMTS4 and HA‐tagged WT‐TSP1 or mutant TSP1 constructs and stimulated with 10 µg/mL LPS for 48 h. Where indicated, recombinant TIMP3 was added with LPS at 100 nM. Media were centrifuged at 1000 × g for 10 min at 4°C. Active and total TGF‐β1 were measured using a Human/Mouse TGF‐β1 ELISA kit (#EK981; LiankeBio). Active TGF‐β1 was measured directly, whereas total TGF‐β1 was determined after acid activation and neutralization. Concentrations were calculated from standard curves, and the active‐to‐total TGF‐β1 ratio was used to assess extracellular TGF‐β1 activation.

### 4.13 Statistical Analysis

Data are presented as mean ± SEM. Normality was assessed using the Shapiro–Wilk test, and homogeneity of variance was evaluated using Levene's test. All statistical tests were two‐sided. For two‐group comparisons, an unpaired two‐tailed Student's *t*‐test was used for normally distributed data, whereas the Mann–Whitney *U* test was used for non‐normally distributed data. For multiple‐group comparisons, one‐way ANOVA with Tukey's post hoc test was used when data were normally distributed and variances were homogeneous; Welch's ANOVA with Tamhane's T2 correction was used when variances were unequal. Non‐normally distributed multiple‐group data were analysed using Kruskal–Wallis test with Dunn's correction. Survival curves were analysed using the Kaplan–Meier method with the log‐rank test. Repeated‐measures or time‐course data were analysed using repeated‐measures ANOVA or two‐way ANOVA followed by appropriate multiple‐comparison tests. *p* < .05 was considered statistically significant.

## Author Contributions

Wan‐Li Jiang, Di Fan and Qing‐Tao Meng contributed to the conception and design of the experiment. Zi‐Long Lu, Zhe‐Wei Zhang, Shi‐yan Wan and Wen Li performed the experiments and collected the data. Zhe‐Wei Zhang, Shi‐yan Wan, Wen Li, Xiao Lu and Xin Xing contributed to the data analysis and interpretation. Zhe‐Wei Zhang, Shi‐yan Wan and Rong Chen drafted and wrote the manuscript. Wan‐Li Jiang, Qing‐Tao Meng and Di Fan provided resources and funding support. All the authors read and approved the final version of the manuscript.

## FUNDING INFORMATION

This study was supported by the General Program of the National Natural Science Foundation of China (grant no.: 82272185).

## Ethical Approval

All animal experiments were performed in accordance with the National Institutes of Health (NIH) Guide for the Care and Use of Laboratory Animals (8th Edition, 2011) and the ARRIVE (Animal Research: Reporting of In Vivo Experiments) guidelines. The protocol was reviewed and approved by the Institutional Animal Care and Use Committee of Renmin Hospital of Wuhan University. Notably, only male mice were used in this study; therefore, the findings may not be generalizable to female mice.

## CONFLICT OF INTEREST STATEMENT

The authors declare no conflicts of interest.

## Supporting information



Supporting Information

Supporting Information

## Data Availability

All datasets generated in this study are available upon request. Publicly available datasets analysed in this work were obtained from the Gene Expression Omnibus (GEO) under accession numbers GSE79962, GSE142615, GSE229925, GSE266124 and GSE207363. The raw MS files and processed analysis results are available from the corresponding author upon reasonable request.
