# Supplementary material for: ADAMTS4 mediates LPS‐induced cardiac injury and myocardial fibrosis through proteolytic cleavage of TSP1
Source: Clin Transl Med. 2026 Aug 5;16(8):e70769. doi: 10.1002/ctm2.70769 (PMC13443130; doi:10.1002/ctm2.70769)
Supplement: Supplementary file 1 [file CTM2-16-e70769-s001.docx]

**ADAMTS4 Mediates LPS-Induced Cardiac Injury and Myocardial Fibrosis Through Proteolytic Cleavage of TSP1**

**Authors:** Zhe-wei Zhang^1†^, Shi-yan Wan^1†^, Wen Li^3†^, Xiao Lu^1†^, Rong Chen^2^, Xin Xing^1^, Wei Lai^1^, Shu-yang Sheng^1^, Mosebetsi, M.M.^1^, Qing-tao Meng^2^, Zi-long Lu^1*^&Di Fan^4*^& Wan-li Jiang^1*^

^1^Department of Thoracic Surgery, Renmin Hospital of Wuhan University, 430060 Wuhan, China.

^2^Department of Anesthesiology, Renmin Hospital of Wuhan University, 430060 Wuhan, China.

^3^Department of Emergency, Renmin Hospital of Wuhan University,430060 Wuhan,China.

^4^Department of Cardiology, Zhongnan Hospital of Wuhan University, 430060 Wuhan, China.

*** Corresponding author:** Zi-long Lu^1*^&Di Fan^4*^& Wan-li Jiang^1*^

**Address for Correspondence:**

Wan-li Jiang, PhD

Department of Thoracic Surgery,

Renmin Hospital of Wuhan University, 430060 Wuhan, China.

Wuhan University at Jiefang Road 238, 430060 Wuhan, China.

Tel.: +86 18627806783. Email：rm003262@whu.edu.cn

**Supplementary Figure S1: ADAMTS4 expression is preferentially induced in cardiac fibroblasts after LPS stimulation and is consistently upregulated in public septic cardiomyopathy datasets.** (**A-C**) Representative Western blot images and quantification of ADAMTS4 protein expression in primary cardiomyocytes (CMs), cardiac fibroblasts (CFs), and endothelial cells (ECs) under NS or LPS stimulation. ADAMTS4 protein expression showed no significant change in CMs (A) or ECs (C), whereas it was markedly increased in CFs after LPS stimulation (B) (n = 6). GAPDH was used as the loading control. (**D**) ADAMTS4 expression in human myocardial samples from GSE79962. Samples from ischemic heart disease and dilated cardiomyopathy groups were not included. (**E**) Adamts4 expression in mouse heart samples from GSE142615. (**F**) Adamts4 expression in mouse left ventricular samples from GSE229925, including sham (n = 4), high-LVEF (LVEF ≥90%; n = 4), low-LVEF (LVEF <65%; n = 3), and normal-LVEF groups (65% ≤ LVEF <90%; n = 4) at 24 h after CLP. (**G**) Adamts4 expression in rat myocardial samples from GSE266124 (GPL31008; control, n = 5; 10 mg/kg LPS, n = 5. Processed expression matrices were analyzed independently after quality control as described in the Methods. Analysis of public GEO datasets showing consistent upregulation of Adamts4 expression in septic cardiomyopathy-related samples across species. (**H**) Serum ADAMTS4 concentrations measured by ELISA in mice following NS or LPS treatment. LPS stimulation significantly increased circulating ADAMTS4 levels compared with the NS group. (**I**) Cardiac tissue ADAMTS4 content measured by ELISA in mice following NS or LPS treatment. LPS stimulation markedly increased cardiac ADAMTS4 content compared with the NS group. Adamts4 expression was increased in human septic samples from GSE79962 (D), LPS-treated rat samples from GSE266124 (E), LPS-challenged mouse samples from GSE142615 (F), and CLP-operated mouse samples from GSE229925 stratified by left ventricular ejection fraction (LVEF) (G). In GSE229925, animals were divided into CLP+Low LVEF (LVEF < 65%), CLP+Mid LVEF (65% ≤ LVEF < 90%), and CLP+High LVEF (LVEF ≥ 90%) groups, with Con indicating sham-operated controls. Data are presented as mean±SEM where applicable. Two-group comparisons were performed using an unpaired two-tailed Student’s t-test or Mann-Whitney U test according to data distribution. Multiple-group comparisons were performed using one-way ANOVA with Tukey’s post hoc test or Kruskal-Wallis test with Dunn’s correction, as appropriate. *P < 0.05; ns, not significant**.**

**Supplementary Figure S2: Col1a2-lineage Adamts4-deficient mice attenuate lipopolysaccharide (LPS)-induced cardiac dysfunction and myocardial fibrosis.** (**A-B**) Col1a2-lineage Adamts4-deficient mice (Adamts4 cfKO) were generated by crossing Adamts4-floxed (Adamts4^fl/fl^) mice with Col1a2-CreER mice. Representative Western blot images and statistical analysis of Adamts4 protein expression in cardiac fibroblasts (CFs) isolated from Adamts4 cfKO mice and littermate controls (n = 6). (**C**) Survival curves of mice challenged with LPS (15 mg/kg) (n = 12). (**D-E**) Dynamic changes in ejection fraction (EF) and fractional shortening (FS) in different groups of mice after LPS stimulation (n = 12). (**F-G**) Changes in plasma levels of lactate dehydrogenase (LDH) and creatine kinase-MB (CK-MB) in different groups of mice after LPS stimulation (n = 6). (**H**) Representative immunofluorescence images showing DHE distribution and quantification of DHE fluorescence intensity (n = 6). (**I**) TUNEL staining and quantification of apoptotic cells in myocardial sections from different groups of mice (n = 6). (**J**) PSR staining showing reduced cardiac fibrosis area in Adamts4 cfKO mice after LPS stimulation (n = 6). Data are presented as mean ± SEM. Normality was assessed using the Shapiro-Wilk test. All tests were two-sided. For two-group comparisons (Figure 4B), an unpaired two-tailed Student's t-test was used. For multiple comparisons (Figures 4D-J), one-way ANOVA with Tukey's post hoc test was applied following Levene's test for homogeneity of variance; Welch's ANOVA with Tamhane's T2 correction was used when variances were unequal. Survival analysis (Figure 4C) was performed using the Kaplan-Meier method with the log-rank (Mantel-Cox) test. Non-normally distributed data were analysed using the Mann-Whitney U test or Kruskal-Wallis test with Dunn's correction. Statistical significance was set at P < 0.05. * P < 0.05 versus littermate Adamts4^fl/fl^ controls; # P < 0.05 compared with the LPS-stimulated Adamts4 cfKO group.

**Supplementary Figure S3: Cardiomyocyte-specific Adamts4 deletion does not significantly alter LPS-induced cardiac dysfunction or myocardial fibrosis.** (**A**) Schematic diagram showing the generation of cardiomyocyte-specific Adamts4 knockout mice (Adamts4 cmKO) by crossing Adamts4^fl/fl^ mice with α-MHC-Cre mice. (**B**) Representative Western blot images and quantification of ADAMTS4 protein expression in cardiomyocytes isolated from Adamts4^fl/fl^ and Adamts4 cmKO mice, confirming efficient Adamts4 deletion in cardiomyocytes (n = 6). GAPDH was used as the loading control. (**C**) Kaplan-Meier survival curves of Adamts4^fl/fl^ and Adamts4 cmKO mice after high-dose LPS challenge (15 mg/kg) (n = 12). (**D-E**) Echocardiographic assessment of ejection fraction (EF) and fractional shortening (FS) in different groups after LPS stimulation (n = 12). (**F-G**) Plasma levels of creatine kinase-MB (CK-MB) and lactate dehydrogenase (LDH) after LPS stimulation (n = 6). (**H**) Representative Picrosirius red (PSR) staining images and quantification of myocardial fibrotic area in Adamts4^fl/fl^ and Adamts4 cmKO mice after LPS stimulation (n = 6). Data are presented as mean±SEM. For panel B, an unpaired two-tailed Student’s t-test was used. For multiple-group comparisons, one-way ANOVA with Tukey’s post hoc test was applied when variances were equal, and Welch’s ANOVA with Tamhane’s T2 correction was used when variances were unequal. Survival analysis was performed using the Kaplan–Meier method with the log-rank test. *P < 0.05 versus Adamts4^fl/fl^ controls for knockout validation; ns, not significant.

**Supplementary Figure S4: Endothelial cell-specific Adamts4 deletion does not significantly alter LPS-induced cardiac dysfunction or myocardial fibrosis.** (**A**) Schematic diagram showing the generation of endothelial cell-specific Adamts4 knockout mice (Adamts4 ecKO) by crossing Adamts4^fl/fl^ mice with Tie2-Cre mice. (**B**) Representative Western blot images and quantification of ADAMTS4 protein expression in endothelial cells isolated from Adamts4^fl/fl^ and Adamts4 ecKO mice, confirming efficient Adamts4 deletion in endothelial cells (n = 6). GAPDH was used as the loading control. (**C**) Kaplan-Meier survival curves of Adamts4^fl/fl^ and Adamts4 ecKO mice after high-dose LPS challenge (15 mg/kg) (n = 12). (**D-E**) Echocardiographic assessment of ejection fraction (EF) and fractional shortening (FS) in different groups after LPS stimulation (n = 12). (**F-G**) Plasma levels of creatine kinase-MB (CK-MB) and lactate dehydrogenase (LDH) after LPS stimulation (n = 6). (**H**) Representative PSR staining images and quantification of myocardial fibrotic area in Adamts4^fl/fl^ and Adamts4 ecKO mice after LPS stimulation (n = 6). Data are presented as mean±SEM. For panel B, an unpaired two-tailed Student’s t-test was used. For multiple-group comparisons, one-way ANOVA with Tukey’s post hoc test was applied when variances were equal, and Welch’s ANOVA with Tamhane’s T2 correction was used when variances were unequal. Survival analysis was performed using the Kaplan-Meier method with the log-rank test. *P < 0.05 versus Adamts4^fl/fl controls for knockout validation; ns, not significant.

**Supplementary Figure S5: Cardiac fibroblast-specific Adamts4 overexpression exacerbates LPS-induced myocardial apoptosis.** (**A**) Representative Western blot images of apoptosis-related proteins, including Bax, Bcl-2, caspase-3, and cleaved caspase-3, in heart tissues from Adamts4 Tg^fl/fl and cardiac fibroblast-specific Adamts4 transgenic mice (Adamts4 cTg) under NS or LPS stimulation. GAPDH was used as the loading control. (**B-D**) Quantification of Bax, Bcl-2, and cleaved caspase-3 protein levels from panel A (n = 6). Bax and cleaved caspase-3 were increased, whereas Bcl-2 was reduced in Adamts4 cTg hearts after LPS stimulation. (**E**) Representative TUNEL staining images and quantification of apoptotic cells in myocardial sections from different groups (n = 6). Data are presented as mean±SEM. Multiple-group comparisons were performed using one-way ANOVA with Tukey’s post hoc test or Welch’s ANOVA with Tamhane’s T2 correction, as appropriate. *P < 0.05 versus the corresponding NS group; #P < 0.05 versus LPS-treated Adamts4 Tg^fl/fl^ mice.

**Supplementary Figure S6: Validation of Adamts4 knockdown and overexpression efficiency in primary cardiac fibroblasts.** (**A**) Representative Western blot images and quantification showing efficient Adamts4 knockdown in primary mouse cardiac fibroblasts transduced with shRNA targeting Adamts4 (shAdamts4) compared with control shRNA (shRNA) (n = 6). (**B**) Representative Western blot images and quantification showing efficient Adamts4 overexpression in primary mouse cardiac fibroblasts transduced with Adamts4-overexpressing vector (oeAdamts4) compared with control vector (oeRNA) (n = 6). GAPDH was used as the loading control. Data are presented as mean±SEM. Two-group comparisons were performed using an unpaired two-tailed Student’s t-test. *P < 0.05.

**Supplementary Figure S7: ADAMTS4 manipulation regulates inflammatory responses and cell viability in LPS-stimulated cardiac fibroblasts, and TSP1 knockdown partially improves cell viability under Adamts4 overexpression.** (**A-B**) RT-qPCR analysis of IL-1β and TNF-α mRNA levels in primary cardiac fibroblasts transduced with shAdamts4 or control shRNA under PBS or LPS stimulation (n = 6). Adamts4 knockdown attenuated LPS-induced inflammatory cytokine expression. (**C-D**) RT-qPCR analysis of IL-1β and TNF-α mRNA levels in primary cardiac fibroblasts transduced with oeAdamts4 or control vector (oeRNA) under PBS or LPS stimulation (n = 6). Adamts4 overexpression enhanced LPS-induced inflammatory cytokine expression. (**E**) Representative Western blot images and quantification showing efficient TSP1 knockdown in cardiac fibroblasts transduced with shTsp1 compared with control shRNA (n = 6). GAPDH was used as the loading control. (**F**) CCK-8 assay showing that shTsp1 improved the viability of cardiac fibroblasts isolated from Adamts4 cTg mice after LPS stimulation (n = 6). (**G**) CCK-8 assay showing that Adamts4 knockdown improved the viability of LPS-stimulated cardiac fibroblasts (n = 6). (**H**) CCK-8 assay showing that Adamts4 overexpression reduced the viability of LPS-stimulated cardiac fibroblasts (n = 6). (**I**) KEGG functional classification of proteins identified by ADAMTS4 Co-IP-MS using heart tissues from LPS-stimulated Adamts4^fl/fl^ mice, showing enrichment of candidate ADAMTS4-associated proteins in cardiovascular disease-related categories. The horizontal axis represents gene counts, and the vertical axis represents second-level KEGG functional classifications. Data are presented as mean±SEM. Two-group comparisons were performed using an unpaired two-tailed Student’s t-test. Multiple-group comparisons were performed using one-way ANOVA with Tukey’s post hoc test or Welch’s ANOVA with Tamhane’s T2 correction, as appropriate. Time-course CCK-8 data were analyzed using two-way ANOVA followed by Sidak’s multiple-comparisons test. *P < 0.05 versus the corresponding PBS or control group; #P < 0.05 versus the corresponding LPS-treated control group.

**Supplementary Figure S8: ADAMTS4 neutralizing antibody reduces TSP1 fragment accumulation in Adamts4-overexpressing cardiac fibroblasts.** (**A**) Representative Western blot images and quantification of full-length TSP1 and TSP1-derived fragments in cardiac fibroblasts with Adamts4 overexpression treated with increasing concentrations of ADAMTS4 neutralizing antibody. Adamts4 overexpression promoted the accumulation of TSP1-derived fragments, whereas ADAMTS4 neutralizing antibody reduced TSP1 fragment abundance in a concentration-dependent manner. Full-length TSP1 in cell lysates was normalized to GAPDH, whereas secreted TSP1-derived fragments in conditioned media were normalized to total protein loading or total membrane protein staining. (B) Representative Western blot images and quantification of TSP1 processing in whole-heart lysates from Adamts4^fl/fl^ mice under basal and LPS-stimulated conditions. Full-length TSP1 was detected at approximately 170 kDa, whereas an approximately 25 kDa TSP1-immunoreactive fragment was increased after LPS stimulation. Both full-length TSP1 and the approximately 25 kDa fragment were detected from total whole-heart lysates and normalized to GAPDH. Because this analysis was performed using whole-heart lysates, it provides tissue-level evidence of TSP1 processing in LPS-stimulated hearts but does not by itself distinguish fibroblast-specific or ADAMTS4-specific TSP1 cleavage. Data are presented as mean±SEM. For panel A, multiple-group comparisons were performed using one-way ANOVA with Tukey’s post hoc test or Welch’s ANOVA with Tamhane’s T2 correction, as appropriate. For panel B, two-group comparisons were performed using an unpaired two-tailed Student’s t-test or Mann-Whitney U test, as appropriate. *P < 0.05 versus the oeAdamts4 group without neutralizing antibody in panel A; *P < 0.05 versus the basal group in panel B.

**Supplementary Figure S9: Prediction and structural prioritization of candidate ADAMTS4 cleavage regions within the N-terminal region of TSP1.** (**A**) Prosperous Plus protease substrate prediction analysis of the N-terminal 400 amino acids of mouse TSP1. High-confidence candidate motifs were identified according to prediction scores. (**B**) Summary of five candidate cleavage regions selected from the prediction analysis, including their amino acid positions, local sequences, and prediction scores. (**C**) AlphaFold-based structural visualization of the five predicted candidate regions. Candidate regions with favorable solvent-exposed accessibility were selected for subsequent mutagenesis analysis, whereas structurally buried or sterically constrained regions were excluded from functional testing. The accessible candidate regions were subsequently renamed according to their order in the TSP1 sequence as Mut1 (236-246 aa), Mut2 (298-308 aa), and Mut3 (322-332 aa) for functional validation in Figure 8. This analysis provided the structural basis for selecting candidate TSP1 cleavage regions for site-directed mutagenesis.

**Supplementary Figure S10: Sanger sequencing validation of TSP1 mutant constructs.** (**A-C**) Representative Sanger sequencing chromatograms confirming successful generation of single-site TSP1 mutants Mut1, Mut2, and Mut3. (**D**) Representative Sanger sequencing chromatograms confirming successful generation of the triple-mutant TSP1 construct Mut1+2+3. Mut1, Mut2, and Mut3 correspond to the cleavage-site candidate regions used for functional validation in Figure 8, including Mut1 at residues 236-246, Mut2 at residues 298-308, and Mut3 at residues 322-332 of mouse TSP1. All constructs were verified by Sanger sequencing before use.

**Supplementary Figure S11: Cleavage-resistant Mut1-TSP1 attenuates ADAMTS4/LPS-induced activation of TGF-β1.** (**A**) ELISA quantification of active TGF-β1 levels in conditioned media from NIH/3T3 fibroblasts co-expressing Flag-ADAMTS4 with either WT-TSP1 or cleavage-resistant Mut1-TSP1 under unstimulated or LPS-stimulated conditions. LPS stimulation increased active TGF-β1 levels in WT-TSP1-reconstituted cells, whereas this effect was reduced in cells reconstituted with Mut1-TSP1. (**B**) ELISA quantification of total TGF-β1 levels in conditioned media from the same experimental groups. Total TGF-β1 was measured after acid activation and neutralization according to the kit protocol. (**C**) Ratio of active TGF-β1 to total TGF-β1, reflecting extracellular TGF-β1 activation. WT-TSP1 reconstitution increased the active/total TGF-β1 ratio under LPS stimulation, whereas Mut1-TSP1 reconstitution blunted this effect. Data are presented as mean±SEM. Multiple-group comparisons were performed using one-way ANOVA with Tukey’s post hoc test or Welch’s ANOVA with Tamhane’s T2 correction, as appropriate. #P < 0.05 versus the corresponding unstimulated WT-TSP1 rescue group; *P < 0.05 versus the LPS-stimulated WT-TSP1 rescue group; ns, not significan
