# Supplementary material for: ADAMTS4 mediates LPS‐induced cardiac injury and myocardial fibrosis through proteolytic cleavage of TSP1
Source: Clin Transl Med. 2026 Aug 5;16(8):e70769. doi: 10.1002/ctm2.70769 (PMC13443130; doi:10.1002/ctm2.70769)
Supplement: Supplementary file 2 [file CTM2-16-e70769-s002.docx]

**Supplementary Western blotting and antibodies**

Western blot quantification was performed using original, unprocessed image files. Representative blot images shown in figures were subjected only to linear, global brightness/contrast adjustments applied uniformly to the entire image, without selective modification of individual bands or lanes.

**Table S1**

| **Target** | **Host** | **Catalog No.** | **Supplier** |
| --- | --- | --- | --- |
| ADAMTS4 | Rabbit pAb | ab185722 | Abcam |
| ADAMTS4 | Rabbit pAb | A2525 | ABclonal |
| TSP1 / THBS1 | Rabbit mAb | #37879 | Cell Signaling Technology |
| α-SMA | Mouse mAb | ab7817 | Abcam |
| α-SMA / ACTA2 | Rabbit mAb / pAb | A17910 | ABclonal |
| GAPDH | Mouse mAb | ab8275 | Abcam |
| GAPDH | Rabbit mAb | A19056 | ABclonal |
| Bcl-2 | Rabbit mAb | ab241548 | Abcam |
| Bax | Rabbit mAb | ab32503 | Abcam |
| Bax | Rabbit pAb | A0207 | ABclonal |
| Caspase-3 | Rabbit mAb | ab184787 | Abcam |
| Caspase-3 | Rabbit pAb | A2156 | ABclonal |
| Cleaved caspase-3 | Rabbit pAb | ab2302 | Abcam |
| Cleaved caspase-3 | Rabbit mAb | A27145 | ABclonal |
| IKKβ | Rabbit pAb | ab38515 | Abcam |
| p-IKKβ | Rabbit pAb | ab194528 | Abcam |
| IκBα | Rabbit mAb | ab92700 | Abcam |
| p-IκBα | Rabbit mAb / pAb | AP0707 | ABclonal |
| NF-κB p65 | Rabbit pAb | ab16502 | Abcam |
| p-NF-κB p65 | Rabbit pAb | ab53489 | Abcam |
| p-NF-κB p65 / RelA-S536 | Rabbit mAb | AP1294 | ABclonal |
| Smad2 | Rabbit pAb | A11498 | ABclonal |
| p-Smad2-S465/S467 | Rabbit mAb | AP1342 | ABclonal |
| HA-tag | Rabbit mAb | AE105 | ABclonal |
| Flag / DDDDK-tag | Rabbit mAb | AE063 | ABclonal |
| HRP-conjugated secondary antibody | Goat anti-rabbit / anti-mouse | #170-6515 | Bio-Rad |
| HRP-conjugated Goat anti-Rabbit IgG | Goat anti-rabbit | AS014 | ABclonal |
| HRP-conjugated Goat anti-Mouse IgG | Goat anti-mouse | AS003 | ABclonal |

**Supplementary RT-qPCR primer sequence**

**Table S2**

| **Gene** | **Species** | **Sequence (5'→3')** | |
| --- | --- | --- | --- |
| Adamts4 | Mouse | Forward | ATGGCCTCAATCCATCCCAG |
|  |  | Reverse | AAGCAGGGTTGGAATCTTTGC |
| Col1a1 | Mouse | Forward | GCTCCTCTTAGGGGCCACT |
|  |  | Reverse | CCACGTCTCACCATTGGGG |
| Col3a1 | Mouse | Forward | CTGTAACATGGAAACTGGGGAAA |
|  |  | Reverse | CCATAGCTGAACTGAAAACCACC |
| α-SMA | Mouse | Forward | GTCCCAGACATCAGGGAGTAA |
|  |  | Reverse | TCGGATACTTCAGCGTCAGGA |
| IL-6 | Mouse | Forward | TAGTCCTTCCTACCCCAATTTCC |
|  |  | Reverse | TTGGTCCTTAGCCACTCCTTC |
| Tnf | Mouse | Forward | CCCTCACACTCAGATCATCTTCT |
|  |  | Reverse | GCTACGACGTGGGCTACAG |
| IL-1β | Mouse | Forward | GCAACTGTTCCTGAACTCAACT |
|  |  | Reverse | ATCTTTTGGGGTCCGTCAACT |
| IL-10 | Mouse | Forward | GCTCTTACTGACTGGCATGAG |
|  |  | Reverse | CGCAGCTCTAGGAGCATGTG |
| MCP1 | Mouse | Forward | TTAAAAACCTGGATCGGAACCAA |
|  |  | Reverse | GCATTAGCTTCAGATTTACGGGT |
| IL-8 | Mouse | Forward | CAAGGCTGGTCCATGCTCC |
|  |  | Reverse | TGCTATCACTTCCTTTCTGTTGC |
| Gapdh | Mouse | Forward | AGGTCGGTGTGAACGGATTTG |
|  |  | Reverse | TGTAGACCATGTAGTTGAGGTCA |

**Public dataset analysis**

Public datasets were downloaded from GEO, including GSE79962, GSE142615, GSE229925, GSE266124, and GSE207363. Bulk expression datasets were normalized according to the original processed matrix files or re-normalized before comparison. *Adamts4* expression was extracted and compared between control and septic cardiomyopathy-related groups. For GSE229925, CLP-operated mice were stratified according to LVEF into Low, Mid, and High LVEF groups. For GSE207363, scRNA-seq data were processed using Seurat; cells with low gene counts, high mitochondrial gene percentage, or poor-quality profiles were removed, followed by normalization, dimensionality reduction, clustering, and cell-type annotation based on canonical marker genes. Dataset accession numbers, species, model types, and sample information are provided in the corresponding figure legends.
